# A unique NLRC4 receptor from echinoderms mediates *Vibrio* phagocytosis via rearrangement of the cytoskeleton and polymerization of F-actin

**DOI:** 10.1371/journal.ppat.1010145

**Published:** 2021-12-13

**Authors:** Kaiyu Chen, Siyuan Zhang, Yina Shao, Ming Guo, Weiwei Zhang, Chenghua Li

**Affiliations:** 1 State Key Laboratory for Managing Biotic and Chemical Threats to the Quality and Safety of Agro-products, Ningbo University, Ningbo, PR China; 2 State-Province Joint Laboratory of Marine Biotechnology and Engineering, Ningbo University, Ningbo, PR China; 3 Laboratory for Marine Fisheries Science and Food Production Processes, Qingdao National Laboratory for Marine Science and Technology, Qingdao, PR China; Uppsala University, SWEDEN

## Abstract

Many members of the nucleotide-binding and oligomerization domain (NACHT)- and leucine-rich-repeat-containing protein (NLR) family play crucial roles in pathogen recognition and innate immune response regulation. In our previous work, a unique and *Vibrio splendidus*-inducible NLRC4 receptor comprising Ig and NACHT domains was identified from the sea cucumber *Apostichopus japonicus*, and this receptor lacked the CARD and LRR domains that are typical of common cytoplasmic NLRs. To better understand the functional role of AjNLRC4, we confirmed that AjNLRC4 was a bona fide membrane PRR with two transmembrane structures. AjNLRC4 was able to directly bind microbes and polysaccharides via its extracellular Ig domain and agglutinate a variety of microbes in a Ca^2+^-dependent manner. Knockdown of AjNLRC4 by RNA interference and blockade of AjNLRC4 by antibodies in coelomocytes both could significantly inhibit the phagocytic activity and elimination of *V*. *splendidus*. Conversely, overexpression of AjNLRC4 enhanced the phagocytic activity of *V*. *splendidus*, and this effect could be specifically blocked by treatment with the actin-mediated endocytosis inhibitor cytochalasin D but not other endocytosis inhibitors. Moreover, AjNLRC4-mediated phagocytic activity was dependent on the interaction between the intracellular domain of AjNLRC4 and the β-actin protein and further regulated the Arp2/3 complex to mediate the rearrangement of the cytoskeleton and the polymerization of F-actin. *V*. *splendidus* was found to be colocalized with lysosomes in coelomocytes, and the bacterial quantities were increased after injection of chloroquine, a lysosome inhibitor. Collectively, these results suggested that AjNLRC4 served as a novel membrane PRR in mediating coelomocyte phagocytosis and further clearing intracellular *Vibrio* through the AjNLRC4-β-actin-Arp2/3 complex-lysosome pathway.

## Author summary

*Vibrio splendidus* is ubiquitously present in marine environments and in or on many aquaculture species and is considered to be an important opportunistic pathogen that has caused serious economic losses to the aquaculture industry worldwide. Phagocytosis is the first step of pathogen clearance and is triggered by specific interactions between host pattern recognition receptors (PRRs) and pathogen-associated molecular patterns (PAMPs) from invasive bacteria. However, the mechanism that underlies receptor-mediated *V*. *splendidus* phagocytosis is poorly understood. In this study, an atypical AjNLRC4 receptor without LRR and CARD domains was found to serve as the membrane receptor for *V*. *splendidus*, not the common cytoplasmic NLRs. The Ig domain of AjNLRC4 is replaced with a conventional LRR domain to bind *V*. *splendidus*, and the intracellular domain of AjNLRC4 specifically interacts with β-actin to mediate *V*. *splendidus* endocytosis in an actin-dependent manner. Endocytic *V*. *splendidus* is ultimately degraded in phagolysosomes. Our findings will contribute to the development of novel strategies for treating *V*. *splendidus* infection by modulating the actin-dependent endocytosis pathway.

## Introduction

Host innate immunity has emerged as the first line of defense against the invasion of endogenous antigens and foreign pathogens throughout long-term phylogenetic evolution [1]. Pattern recognition receptors (PRRs) are critical parts of innate immunity and play important roles in initiating innate immune responses by recognizing a variety of pathogen- and danger-associated molecular patterns (PAMPs and DAMPs, respectively), such as lipopolysaccharide (LPS), peptidoglycan (PGN), flagellin, mannan (MAN) and danger signals, including uric acid, ATP, and HMGB1 [2]. After recognizing these molecules, PRRs activate downstream signaling pathways to generate the corresponding immune responses [3]. Several PRR families with different subcellular localizations, such as Toll-like receptors (TLRs), C-type lectin receptors (CLRs), RIG-I-like receptors (RLRs), HIN-200 proteins and nucleotide-binding and oligomerization domain-like receptors (NLRs), have been characterized [4–8]. Among these families, the largest is the NLR family, which has attracted substantial attention due to the roles of these proteins as intracellular receptors of PAMPs and DAMPs.

NLRs share a typical three-part domain architecture, which includes a C-terminal LRR domain, a NACHT core domain, and an N-terminal effector domain. The cooperation of each domain enables NLRs to activate themselves and mediate downstream immune responses. The LRR domain at the C-terminus is mainly responsible for recognizing and binding to PAMPs and DAMPs and then mediating the interaction between NLRs and downstream adaptor proteins to activate immune cascades [9–10]. For example, both NOD1 and NOD2 can recognize partial structures of bacterial peptidoglycans, γ-D-glutamyl-meso-diaminopimelic acid and muramyl dipeptides by their LRR domains [11–13]. However, NLRP10 is the only NLR protein that does not contain LRRs. This circumstance suggests a possible function of NLRP10 as an adaptor or regulatory protein rather than as a sensor of PAMPs or DAMPs [14]. The NACHT domain is the only domain that is common to all NLR family members and is composed of four substructures: neuronal apoptosis inhibitory protein (NAIP), class II transactivator (CIIT), incompatibility locus protein from *Podospora anserina* (HET-E) and telomerase-associated protein (TP1). This domain enables the activation of the signaling complex via ATP-dependent oligomerization, which is essential for the downstream functions of several NLRs [15–18]. In addition, the NACHT domain can directly interact with other proteins to form a network that potentially regulates the immune response against invading pathogens. For instance, NLRX1 from black carp directly inhibits mitochondrial antiviral signaling protein (MAVS)-mediated antiviral signaling through the interaction between the NACHT domain and MAVS [19]. Based on the differences in their N-terminal domains, NLRs can be divided into five subfamilies, namely, NLRA with an AD (acidic transactivation domain) domain, NLRB with a BIR (baculovirus inhibitor of apoptosis protein repeat domain) domain, NLRC with a CARD (caspase activating and recruitment domain) domain, NLRP with a PYD (pyrin domain) domain, and NLRX with an unknown domain [20]. CIITA, the only member of the NLRA family, is a positive regulator of the transcription of MHC class II molecules that transports proteins to the nucleus [21], and NAIP, the only member of the NLRB family, prevents apoptosis by inhibiting the activities of CASP3, CASP7 and CASP9 [22,23]. The NLRP subfamily consists of 14 members, which contains a variety of sequence motifs conserved in human proteins to mediate apoptosis and inflammatory signal transduction [9,24]. The N-terminal effector domain is mainly responsible for the recognition of adaptor molecules and downstream effectors and mediates and activates downstream NLR signal transduction through homotypic domain interactions. The NLRC subfamily consists of three members: NOD1, NOD2, and NLRC4. These proteins are monitors of microbial invasion and the immune system and mainly affect the secretion of cytokines by activating or inhibiting signaling pathways. The NLRC3, NLRC5 and NLRX1 members are classified into the NLRC subfamily due to their homology and phylogenetic relationships, although their N-terminal domains have not yet been fully characterized [25]. These subfamily members participate in the initiation of autophagy and regulate the inflammatory response and the activity of type I IFN signaling pathways [26]. NLRC4 can bind to the ASC protein through homotypic CARD-CARD interactions and recruit pro-caspase-1 to form a protein complex capable of activating caspase-1. On the other hand, NLRC4 directly uses its N-terminal CARD domain to associate with the CARD domain of pro-caspase-1 to activate caspase-1.

In our previous work, a novel NLR gene was identified from transcriptome analysis in sea cucumber [27], an economical aquaculture species in East Asia. Unlike other conventional NLRs, this receptor is composed of a NACHT domain characteristic of NLRs and an Ig domain characteristic of Ig domain-containing proteins (ICPs) but lacks LRR and CARD domains. The Ig domain, with approximately 80 amino acids, is considered to be one of the most widely distributed domains in animals [28] and consists of 7 to 9 antiparallel β-sheets by 3D topological structure analysis. The two β-sheets are connected through disulfide bonds to form a β-sandwich structure [29]. The topological features of the Ig domain enable ICPs to be widely involved in various fundamental processes, such as cell adhesion [30], neural recognition [31], interactions between cytokines and their receptors [32], and, more importantly, immune responses. Numerous ICPs with one or more Ig domains have been identified in invertebrates [25,33]. Ig domain-containing protein junctional adhesion molecule A (CgJAM-A-L) and CgCAICP-1 are located on the hemocyte membrane, functioning as PRRs to recognize different bacteria and facilitating the phagocytosis of *V*. *splendidus* in *C*. *gigas* [34]. It has also been found that some invertebrate ICPs undergo alternative splicing after immune stimulation, which is in contrast to the Ig-(and TCR) recombination of antibody molecules in vertebrates [35]. For example, in the Chinese mitten crab *Eriocheir sinensis*, EsDscam can generate 30,600 isoforms through the alternative splicing of three Ig domains, and these isoforms can specifically recognize different bacteria [36]. In contrast to invertebrate Dscam, the equivalent in vertebrates is Ig-(and TCR) recombination, not alternative splicing. All of these results indicate that diverse ICPs are created by the rearrangement or alternative splicing of the Ig domain and enable specific recognition against bacteria [37]. In invertebrates, ICPs function as PRRs and participate in the innate immune response, such as in the processes of recognition, phagocytosis, nodule formation, encapsulation, and effector synthesis [38–40]. For instance, in the crayfish *Procambarus clarkii*, a novel C-type lectin (PcLec3) can bind to invading bacteria with its CTLD and Ig domains and subsequently enhance hemocyte adhesion and phagocytosis via the Ig domain [41]. This evidence supports that the Ig domain is the basic recognition module of typical PRRs when participating in immune recognition.

Ig domains and NACHT domains are the characteristic domains of ICPs and NLRs for immune recognition and signal transduction, respectively. However, proteins that contain both Ig and NACHT domains have not been reported to our knowledge, and immune functions are not well documented. This study will conduct the functional elucidation of sea cucumber NLR receptors that contain both Ig domains and NACHT domains, focusing on the molecular mechanism that mediates coelomocyte phagocytic activity.

## Materials and methods

### Animals

Healthy adult sea cucumbers (*A*. *japonicus*) weighing 125 ± 15 g were collected from the Dalian Pacific Aquaculture Company (Dalian, China) and temporarily maintained in 30 L of aerated natural seawater (salinity of 28 ± 1, temperature of 16 ± 1°C) for two days. Sea cucumbers were randomly selected for the following experiments.

### Recombinant expression of AjNLRC4 and production of AjNLRC4 antiserum

The specific primers AjNLRC4-EX-F and AjNLRC4-EX-R (Table 1) were used to amplify the extracellular fragment of AjNLRC4. Recombinant AjNLRC4-EX was generated according to our previous work [27,42]. The recombinant AjNLRC4–EX protein was purified using a Ni-NTA Sepharose column (Roche) and dialyzed three times at 4°C to recover the protein activity. The concentration of the recombinant protein was quantified using the bicinchoninic acid method (Sangon Biotechnology, Shanghai). Mouse antiserum against AjNLRC4 was prepared following a previously reported method [43]. Similarly, the sequences of AjNLRC4-IN and Ajβ-actin (BAH79732.1) were also amplified with the primers in Table 1 and cloned into the pET-28a^+^ vector to generate recombinant proteins. Purified His-tagged AjNLRC4-IN and His-tagged Ajβ-actin were used for further functional analysis.

**Table 1:** Primers used in this study.

Primer	Sequence(5’-3’)	Used for
*Aj*NLRC4-EX F	GGATCCGGGACGCCACAATACCTTGAGCTTG	Recombinant expression
*Aj*NLRC4-EX R	CTCGAGGCAACCACTGACAACTGGAAACGCA	
*Aj*NLRC4-IN F	GGATCCTTTGGTCCAGGTGGACCTCCACCTC	
*Aj*NLRC4-IN R	GCGGCCGCAGGGAGGTTAGGACGCTGACCCATT	
*Aj*β-actin F	GGATTCATGGAGGATGAAGTACAAGCTCTC	
*Aj*β-actin R	CTCGAGTTAGAAGCATTTCCTGTGGACAATG	
M13 F	GTCGTGACTGGGAAAACCCTGGCG	Sequencing
M13 R	GAGCGGATAACAATTTCACACAGG	
*Aj*Arpc1 qF	CCATTCGTTCTACTGTTACCTGCC	Real-time PCR
*Aj*Arpc1 qR	ACCTCCAAATTCCTGCATCACC	
*Aj*Arpc2 qF	AATAGGATCATCGAGGAGAC	
*Aj*Arpc2 qR	CAATGAGGCACTGAGTAGAA	
*Aj*Arpc3 qF	GCTCGGAATTGCTAGTTTCA	
*Aj*Arpc3 qR	GTCGCAGGGTCCATTACTTT	
*Aj*Arpc4 qF	CAGCGACATTACGACCTTAC	
*Aj*Arpc4 qR	ATGACCGGCTTTAACAACAG	
*Aj*Arpc5 qF	CTTGCTAATCCACCAGCCAGTA	
*Aj*Arpc5 qR	ACGCCCTTCTCAACTTCG	
*Aj*NLRC4 qF	AAAGCCAATCTCGAAGAACAGG	
AjNLRC4 qR	ACGAAAGTCGCCGTCAACAC	
*Aj*β-actin qF	CCATTCAACCCTAAAGCCAACA	
*Aj*β-actin qR	ACACACCGTCTCCTGAGTCCAT	
si*Aj*NLRC4-1	CCUUAUUACCUCAUCGAAUTT	RNA interference
si*Aj*NLRC4-2	AUUCGAUGAGGUAAUAAGGTT	
si*Aj*Arpc4-1	GGUUGUACUUGCUUGACUUTT	
si*Aj*Arpc4-2	AAGUCAAGCAAGUACAACCTT	
siRNA-NC (Negative control)	UUCUCCGAACGUGUCACGUTT	
	ACGUGACACGUUCGGAGAATT	

### PAMP binding assay

PAMP binding activity was examined via enzyme-linked immunosorbent assays, as previously described [27]. Three types of PAMPs, namely, LPS (*Escherichia coli* 055:B5; Sigma), PGN (*Staphylococcus aureus*; Sigma), and MAN (*Saccharomyces cerevisiae*; Sigma), were used, and another type of LPS was isolated from *V*. *splendidus* using an LPS extraction kit (Beibokit, China) following the manufacturer’s instructions. These PAMPs were separately dissolved in carbonate–bicarbonate buffer (50 mmol L^−1^, pH 9.6) at a final concentration of 0.2 mg mL^−1^. Then, 30 μL of each PAMP solution was placed into a 96-well plate and incubated at 4°C overnight before washing three times with phosphate-buffered saline (PBS) with 0.05% Tween-20 (PBST) at pH 7.2. The wells were blocked with 200 μL 5% BSA in PBS for 1 h at 37°C and washed three times with PBST. Subsequently, 100 μL rAjNLRC4-EX protein was added at different doses (0, 2.5, 5, 7.5, 10, 12.5, 15, 17.5 and 20 μg), and the plate was incubated at 37°C for 1 h. Each well was washed twice and then incubated with 100 μL mouse anti-His tag monoclonal antibody (1:1000 dilution). The samples were incubated for 1 h at 37°C, 100 μL AP-labeled goat anti-mouse IgG (1:3000 dilution) was added, and the samples were incubated again at 37°C for 1 h. Thereafter, the samples were washed four times with PBST, 100 μL PNPP chromogenic substrate reagent (Beijing Seitz Biotechnology Company) was added, and the samples were incubated in the dark at room temperature for 30 min. The chromogenic reaction was stopped by adding 50 μL 3 mol L^−1^ NaOH to each well, and the absorbance was measured at a wavelength of 450 nm using a microplate reader (Thermo Fisher Scientific). The same concentrations of BSA (0, 2.5, 5, 7.5, 10, 12.5, 15, 17.5 and 20 μg) were used as controls, and Tris-buffered saline (50 mmol L^−1^ Tris-HCl, 50 mmol L^−1^ NaCl, pH 7.5) without any exogenous protein served as the blank control. Each sample was analyzed in five replicates, and the data are presented as the mean ± SD (n = 5).

### Microbial binding assay

The bacterial binding assays were performed according to a previously described method [44] with slight modifications. Briefly, G^+^ bacteria (*Micrococcus luteus*) and G^–^bacteria (*Vibrio harvey*, *Vibrio parahemolyticus*, *V*.*splendidus* and *E*. *coli*) were used to test the binding activity of rAjNLRC4-EX (Ig-like domain and Ig domain) and rAjNLRC4-IN (NACHT domain) of AjNLRC4. The bacteria were cultured in the corresponding medium overnight and then harvested by centrifugation at 6000 g for 5 min. After rinsing with TBS four times, the bacteria were resuspended in TBS, and the density was adjusted to A_600_ = 1.0. Purified rAjNLRC4-EX and rAjNLRC4-IN (200 μL, 1 mg/ml) were mixed with 200 μL of bacteria and incubated at room temperature with rotation for 1 h. Then, the bacteria were harvested by centrifugation at 6000 g for 5 min and washed four times with TBS. The last eluate and the bacterial precipitate were subjected to 12% SDS–PAGE. The proteins in the gel were transferred to nitrocellulose membranes for western blotting analysis using an anti-His Ab (1:1000 dilution in TBST containing 3% nonfat milk) and an alkaline phosphatase-conjugated horse anti-mouse IgG antibody (1:2000 dilution in TBST containing 5% nonfat milk) as the primary and secondary antibodies, respectively.

### Microbial agglutination assay

*V*. *harvey*, *V*. *parahemolyticus*, *V*. *splendidus*, *E*. *coli* and *M*. *luteus* were used again for the agglutination tests. Microbes were harvested in the mid-logarithmic growth phase, heat killed (75°C for 30 min), washed with 0.1 M NaHCO_3_ (pH 9.0) and stained with FITC (Sigma; 1 mg/ml in 0.1 M NaHCO_3_) at 37°C for 2 h with gentle shaking [45]. The FITC-labeled microbes were washed three times with PBS to eliminate all of the free FITC and resuspended in PBS at a density of A_600_ = 1.0. Fifty microliters of microbe suspension was mixed with 50 μL of rAjNLRC4-EX (1 mg/ml) in the presence or absence of 10 mM CaCl_2_. The mixtures were incubated at room temperature for approximately 2 h. BSA instead of r rAjNLRC4-EX was used as the negative control. LPS (1 mg/mL) and PGN (1 mg/mL) were used for the agglutination inhibition experiment to determine the binding specificity of G^-^ bacteria and G^+^ bacteria, respectively. The Ca^2+^ chelator EDTA (10 mM) was used as another control. The agglutination activities were analyzed using a fluorescence microscope (Nikon), and three independent fields of view were imaged.

### Oligomerization assay

Subric acid bis sodium salt (3-sulfo-N-hydroxysuccinimide ester, BS3; Sigma–Aldrich, USA) was used for cell-surface protein crosslinking. A crosslinking assay was performed *in vivo* to detect oligomerization according to the manufacturer’s protocol. Coelomocytes from sea cucumber were collected and washed three times with ice-cold TBS. BS3 was then added to the resuspended coelomocytes at a final concentration of 5 mM, and the reaction mixture was incubated at 4°C for 1 h. The mixture was terminated by adding SDS–PAGE loading buffer and then treated in boiling water for 8 min followed by SDS–PAGE and western blotting analysis. rAjNLRC4-EX was analyzed by native PAGE to detect oligomerization *in vitro*, as described in previous articles [46].

### Immunocytochemical analysis

To detect the distribution and translocation of AjNLRC4 in the coelomocytes of sea cucumbers challenged with *V*. *splendidus*, immunocytochemical assays were performed following the method described in a previous report [47]. Coelomocytes from *V*. *splendidus*-challenged sea cucumbers were collected in 4% paraformaldehyde and anticoagulant solutions (1:1). The coelomocytes were then washed three times with PBS and centrifuged at 800 × g for 6 min at 4°C to remove the plasma. Then, the cells were resuspended in Leibovitz’s L-15 cell culture medium (Invitrogen, USA) supplemented with penicillin (100 U mL^−1^) and streptomycin sulfate (100 mg mL^−1^) at a final concentration of 1 × 10^6^ cells mL^−1^. NaCl (0.39 M) was added to adjust the osmotic pressure to 780 mmol L^−1^. The treated coelomocytes were dropped onto polylysine-coated glass slides and incubated for 1 h. The slides were washed six times and blocked with 3% bovine serum albumin (dissolved in PBS) for 30 min at 37°C. After that, anti-AjNLRC4 antibody was added (1:100 diluted in 3% bovine serum albumin) and incubated overnight at 4°C. The coelomocytes were washed six times with PBS, incubated with goat anti-mouse antibodies conjugated with Cy3 or Alexa 488 (1:1000 diluted in PBS) for 2 h at 37°C, washed with PBS again, and then stained with 4-6-diamidino-2-phenylindole (DAPI) for 10 min at room temperature. After washing six times, the slides were examined under a laser scanning spectral confocal microscope (TCS SP2; Leica, Germany).

### Coelomocyte binding activity of rAjNLRC4-EX and rAjNLRC4-IN

rAjNLRC4-EX and rAjNLRC4-IN were dialyzed in CFS and then separately injected into sea cucumbers. Coelomocytes were collected 60 min after injection, and the coelomocytes were isolated according to the method described above. The coelomocytes were analyzed by immunohistochemistry as described above. An anti-His tag antibody (1:1000 in PBS containing 3% BSA) was used to detect whether the recombinant proteins could bind to the surfaces of coelomocytes, and FITC-conjugated goat anti-mouse IgG (1:2000 in PBS containing 3% BSA), which could be visualized under laser scanning confocal microscopy (TCS SP2; Leica, Germany), was used as the secondary antibody.

### Colocalization analysis of *V*. *splendidus*, AjNLRC4 and F-actin

To detect the interaction between *V*. *splendidus* and AjNLRC4, *V*. *splendidus* was labeled by incubation with FITC reagent (25 μg/ml, Beyotime, Shanghai, China) for 2 h at 37°C. Then, the mixture was centrifuged at 12000 × g for 20 min at 4°C to remove the supernatant, and the pellet was resuspended in PBS, washed twice with PBS and resuspended in PBS. One hundred microliters of FITC-labeled *V*. *splendidus* (10^6^ CFU/mL) was injected into sea cucumber through a syringe, and the coelomocytes were collected at different time points. TRITC-conjugated phalloidin (Yeasen) was used to stain F-actin in sea cucumber coelomocytes following the manufacturer’s protocol. The cells were subjected to immunocytochemical assays using anti-AjNLRC4 antibodies to detect the colocalization of *V*. *splendidus* and AjNLRC4. A DyLight 649 AffiniPure goat anti-mouse IgG (red) secondary antibody was used to detect AjNLRC4. The nuclei were stained with DAPI (blue). The cells were subjected to immunocytochemical assays using anti-AjNLRC4 antibodies to detect the colocalization of *V*. *splendidus* with AjNLRC4 and F-actin.

### Co-localization of fluorescently labelled *V*. *splendidus* and lysosomes

LysoTracker Red (Beyotime) was used to stain the lysosomes in sea cucumber coelomocytes following the manufacturer’s protocol. These collected coelomocytes were incubated with LysoTracker Red (1:20000 diluted in TBS) and one hundred microlitres FITC-labelled *V. splendidus* (10^6^ CFU/mL) at 16°C for 3 h. The coelomocytes were collected and spread onto slides for observation under a laser scanning spectral confocal microscope.

### RNA interference and antibody blocking assays

siRNAs specific to AjNLRC4 were designed and synthesized by GenePharma (Shanghai, China) (Table 1). Another siRNA (negative control, NC) that was not specific for any unigenes in the *A*. *japonicus* transcriptome served as a negative control. These siRNAs were then dissolved in RNase-free water to generate 20 μM working solutions. For RNA interference, 10 μL AjNLRC4 siRNA and an equal volume of transfection reagent were mixed with 80 μL of phosphate-buffered saline (PBS) to prepare the transfection solution. Each sea cucumber (approximately 100 g in weight) was injected with 100 μL of the transfection solution described above by tentacle injection. The control group was injected with NC siRNA under the same conditions. At 24 h posttransfection, control and treated coelomocytes were harvested to assess the silencing efficiency. At 48 h posttransfection, primary coelomocytes were harvested to conduct western blotting analysis. The treated and negative control groups were set up in triplicate. A similar method was used for the AjArpc4 knockdown assay.

The preserum and anti-AjNLRC4 serum were purified from mice as previously described [27]. Each sea cucumber was injected with 30 μg of purified antibodies, incubated for 2 h, and then injected with FITC-labeled *V*. *splendidus* and incubated for 3 h. A total of 10,000 coelomocytes were acquired to quantify phagocytosis by flow cytometry. There were three replicates in each group.

### AjNLRC4 overexpression

To assess the immune function of AjNLRC4, an AjNLRC4 overexpression assay was performed according to the method of Yang et al [46]. The AjNLRC4 open reading frame (ORF) was amplified using the AjNLRC4-ORF-F and AjNLRC4-ORF-R primers (Table 1). The PCR fragments were then ligated into the pET-32a^+^ vector, which contains a T7 promoter. Thereafter, the recombinant plasmid was used for mRNA synthesis and capping as previously described [46]. The mRNA from the empty pET-32a^+^ vector was used as a control. Each group was injected with 300 μg mRNA and incubated for 24 h, and the overexpression efficiency was detected using AjNLRC4 antibodies. Later, FITC-labeled *V*. *splendidus* was injected into the sea cucumbers for an additional 3 h. Thereafter, the coelomocytes were obtained by centrifugation at 500 g for 5 min, washed and resuspended in PBS. Phagocytic activity, which was the ratio between phagocytic cells and total cells, was detected by flow cytometry. Extracellular fluorescence was quenched by adding 1 ml of 0.5% trypan blue to the cell suspension. The experiments were repeated three times.

### Specific inhibitor assay

Pharmacological inhibitors were often used to investigate the endocytic mechanism responsible for the cellular uptake of particles [48]. To explore the endocytic pathway by which *V*. *splendidus* was taken up as well as the AjNLRC4-mediated endocytic pathway by which *V*. *splendidus* was taken up, we used five specific inhibitors, chlorpromazine (CPZ, clathrin-dependent endocytic pathway inhibitor, Sangon Biotech, Shanghai, China), nystatin (caveolin-dependent endocytic pathway inhibitor, Sangon Biotech, Shanghai, China), IPA-3 (macropinocytosis pathway inhibitor, Aladdin Shanghai, China), cytochalasin D (actin-dependent endocytic pathway inhibitor, Aladdin Shanghai, China), and mitmab (dynamin-dependent endocytic pathway inhibitor, Aladdin Shanghai, China). Coelomocytes were treated with various concentration gradients of these inhibitors for 24 h. Then, cell viability was detected with the MTT kit following the manufacturer’s protocol. A control group was treated with only DMSO or PBS at the same time, and the appropriate working concentrations of the five inhibitors that did not affect the cell viability were determined. After that, coelomocytes were treated with the indicated inhibitor concentrations for 3 h and before injection with one hundred microliters of FITC-labeled *V*. *splendidus* (10^6^ CFU/mL) for another 3 h. The phagocytic activity was determined by flow cytometry. To investigate the AjNLRC4-mediated pathway of *V*. *splendidus* endocytosis, AjNLRC4 was overexpressed by transfection of synthesized AjNLRC4 mRNA *in vivo*, and then, different inhibitors were added to assess phagocytic activity by flow cytometry.

### Cell proliferation inhibition assay

Hydroxyurea (Beyotime, China) was a type of cell proliferation inhibitor that could restrain the synthesis of NDA. To minimize the effect of the change in the proportion of phagocytic cells in the total coelomocytes, we investigated the phagocytic activity of coelomocytes treated with hydroxyurea. Hydroxyurea was diluted to a final concentration of 10 μg/μL with PBS, and 20 μl hydroxyurea solution was injected into sea cucumbers for 6 h before FITC-labeled *V*. *splendidus* injection. The same volume of PBS served as a control. The phagocytic activity was determined by flow cytometry as described below.

### Flow cytometry assay phagocytic activity

*V*. *splendidus* was labeled with FITC (green) for 2 h and then collected by centrifugation at 12000 × g for 20 min. FITC-labeled *V*. *splendidus* was washed twice with PBS and then suspended in PBS for sea cucumber injection experiments. Coelomocytes were harvested 3 h after AjNLRC4 overexpression or knockdown, and phagocytic activity was detected using flow cytometry (ImageStreamX MarkII, USA). A total of 10,000 cells were acquired to quantify the percentage of phagocytic activity. Extracellular fluorescence was quenched by adding 1 ml of 0.5% trypan blue to the cell suspension. The experiments were repeated three times.

### Internalization assay

The internalization assay was performed as previously described [49]. Briefly, *V*. *splendidus* cells were collected at OD_600_ = 1.0, washed with phosphate-buffered saline (PBS, 0.01 M sodium phosphate, 0.8% NaCl, pH 7.2) and resuspended in internalization medium (IM; low-glucose Dulbecco’s modified Eagle’s medium (Gibco) without 10% (v/v) fetal bovine serum (HighClone) and antibiotics). One milliliter of 10^6^ CFU mL^-1^ *V*. *splendidus* suspension was separately added to coelomocyte monolayers in 24-well plates at a final concentration of approximately 10^6^ CFU mL^-1^. After coincubation for 3 h, 6 h and 9 h, each well was washed with IM three times, and one portion of coelomocytes was detached by adding trypsin-like enzyme (TrypLe Express, Gibco) followed by Triton X-100 (0.025%) to release the intracellular bacteria. The other portion of coelomocytes was incubated for another 1.5 h in IM with 500 μg gentamicin (Gibco) per well to kill extracellular *V*. *splendidus* cells. The wells were washed three times, and trypsin-like enzyme and Triton X-100 were sequentially added to release the internalized bacteria. The number of bacteria in both lysates was determined by plate counts.

### Pull-down assay

A pull-down assay was performed to identify AjNLRC4-interacting proteins with recombinant AjNLRC4-EX and AjNLRC4-IN. These different domains were separately amplified using the primers listed in Table 1 and designated AjNLRC4-EX and AjNLRC4-IN. AjNLRC4-IN was separately ligated into the pGEX-4T-1 vector (GE Healthcare) and transformed into *E*. *coli* Rosseta. The recombinant expressed proteins were purified by affinity chromatography using GST resin (GenScript, Nanjing, China). A GST pull-down assay was performed to identify AjNLRC4-IN-interacting proteins. Recombinant GST-AjNLRC4-IN was captured with GST Sefinose Resin for the GST pull-down assay following the manufacturer’s instructions (C600913, Sangon Biotech). The generated beads were incubated with protein extracts from coelomocytes overnight at 4°C. An equal volume lysate solution served as a control. The mixture was subsequently washed three times with 5–10 resin-bed volumes of binding/wash buffer and centrifuged at 700×g for 2 min, and the supernatant was removed. Finally, a resin-bed volume of elution buffer was used to collect the binding proteins by centrifuging the samples three times for 2 min at 700×g. The eluted proteins were analyzed by SDS–PAGE to identify the proteins interacting with AjNLRC4-IN and to compare those proteins with the control samples. The bands that were identified in the AjNLRC4-IN sample but not in the control sample were excised for further analysis by LC-MS/MS. The AjNLRC4-IN interacting proteins were recombinantly expressed in *E*. *coli* using the pET-28a^+^ expression vector. Purified GST-tagged AjNLRC4-IN (200 μg) was separately incubated with the four His-tagged AjNLRC4-IN interacting proteins (1:1) for 5 h at 4°C. After incubation with GST-bound resin (50 μl) for 45 min at 4°C, the resin was washed five times with PBS. Elution buffer (10 mM reduced glutathione, 50 mM Tris-HCl, pH 8.0) was added to elute the bound proteins. SDS–PAGE was conducted to analyze the proteins. His pull-down assays were performed. Purified His-tagged AjNLRC4-IN interacting proteins were incubated with GST-tagged AjNLRC4-IN. After incubation with His-bound resin for 45 min at 4°C, the resin was washed five times with PBS. Elution buffer (0.5 M NaCl, 1 M imidazole, 20 mM Tris-HCl, pH 8.0) was used to elute the bound proteins.

### Microscale thermophoresis (MST) analysis

The binding kinetics of recombinant GST-AjNLRC4-IN to His-β-Αctin were measured by microscale thermophoresis in a Monolith NT. A label-free instrument (Nano Temper Technologies GMBH, Germany) was used to detect changes in the size, charge and conformation induced by binding. Labeled GST-AjNLRC4-IN (10 μM) was displaced by a buffer containing 50 mM Tris-HCl (pH 7.4), 150 mM NaCl, 10 mM MgCl2 and 0.05% (V/V) Tween-20. A range of concentrations of His-β-Actin in the assay buffer (50 mM Tris-HCl pH 7.8, 150 mM NaCl, 10 mM MgCl_2_, 0.05% Tween-20) was incubated with labeled protein (1:1, v/v) for 10 minutes. The sample was loaded into the NT. Label-free standard capillaries were measured with 20% LED power and 80% MST power. The KD Fit function of Nano Temper Analysis Software (Version 1.5.41) was used to fit the curve and calculate the value of the dissociation constant (Kd).

### F-actin to G-actin ratio determination

The F-actin to G-actin ratio was determined by western blotting, as previously described [50]. Briefly, the two forms of actin differed in that F-actin was insoluble, whereas G-actin was soluble. Coelomocytes were collected from the control, siNLRC4 and CK666 inhibitor treatment groups after 3, 6 and 9 h of *V*. *splendidus* infection. Equal numbers of coelomocytes were harvested in cold lysis buffer (10 mM K_2_HPO_4_, 100 mM NaF, 50 mM KCl, 2 mM MgCl_2_, 1 mM EGTA, 0.2 mM DTT, 0.5% Triton X-100, 1 mM sucrose, pH 7.0) and centrifuged at 15,000 g for 30 min. The amount of soluble actin (G-actin) in the supernatant was measured. The insoluble F-actin in the pellet was resuspended in lysis buffer plus an equal volume of buffer 2 (1.5 mM guanidine hydrochloride, 1 mM sodium acetate, 1 mM CaCl_2_, 1 mM ATP, 20 mM Tris-HCl, pH 7.5) and incubated on ice for 1 h with gentle mixing every 15 min to convert insoluble F-actin into soluble G-actin. The samples were centrifuged at 15,000 g for 30 min, and the amount of F-actin in this supernatant was measured. Samples from the supernatant (G-actin) and pellet (F-actin) fractions were proportionally loaded and analyzed by western blotting using an actin-specific antibody (#MAB1501, 1:10,000, Millipore).

### Quantitative real-time PCR analysis

The transcripts of *AjNLRC4* and its coordinator genes were analyzed via quantitative real-time PCR (qRT-PCR) on a Applied Biosystem 7500 real-time PCR system. According to the manufacturer’s protocol, total RNA were extracted with the TRIzol reagent (Takara, Japan), and cDNA was prepared using PrimeScript RT reagent with gDNA Eraser Kit (Takara, Japan). Amplification was conducted in a 20uL reaction volume containing 8 uL of 1:50 diluted cDNA, 0.8 uL of each primer (listed in Table 1), 10 uL of SYBR Green, and 0.4 uL of ROX (Takara, Japan). The reaction mixtures were incubated for 2 min at 95°C, followed by 40 cycles of 15 s at 95 °C, 15 s at 60 °C, and 20 s at 72 °C followed by a melting curve. The baseline was automatically set by the software to maintain consistency. The relative expression levels were calculated using the 2^−ΔΔCT^ method with β-actin for normalization ([51], S1 Text), Each PCR trial was run in triplicate parallel reactions and repeated three times. The primer efficiency was checked. A significant difference in expression relative to expression in the control group at each time point is indicated using an asterisk for *p* < 0.05 and two asterisks for *p* < 0.01.

### Western blotting

The protein concentrations of coelomocyte lysates were determined with a BCA Protein Assay Kit (Sangon). Approximately 50 μg of protein was separated with 10% SDS-polyacrylamide gels and transferred to 0.45μm ECL membranes. After blocking with 5% skim milk in TBST (50 mmol L^−1^ Tris-HCl, 150 mmol L^−1^ NaCl, and 1% Tween-20) at room temperature for 120 min, the membranes were incubated with antibodies diluted at 1:500 in 5% BSA solution at 4°C overnight. The AjNLRC4 antiserum was prepared as described above, the Transferrin, dynamin, caveolin and Rab5 antiserum were were purchased from (Abcam). The membranes were incubated with HRP-labelled anti-rat or mouse IgG (1:2000) in 5% BSA solution at room temperature for 1.5 h. The membranes were incubated in Western Lightning-ECL substrate (Perkin-Elmer) prior to exposure with X-OMAT AR X-ray film (Eastman Kodak, Rochester, NY). The densities of the protein bands were quantified using the ImageJcngr software package, and the results were derived from the statistical analysis of three independent experiments.

### Bacterial clearance assay

One hundred microliters of fresh *V*. *splendidus* (10^6^ CFU/mL) was injected into different groups of sea cucumbers: the AjNLRC4 silencing group, AjNLRC4 silencing + AjNLRC4 mRNA group, AjNLRC4 silencing + Trx-His mRNA group, NC siRNA group, and PBS group. After infection for 12 h, the coelomic fluid was collected from each group and then diluted 10,00 times. One hundred microliters of dilution from each group was plated onto 2216E agar plates and incubated at 28°C overnight. The number of bacteria was determined 24 h later using the plate counting method.

## Results

### AjNLRC4 localized to the membranes of sea cucumber coelomocytes

**Fig 1:**
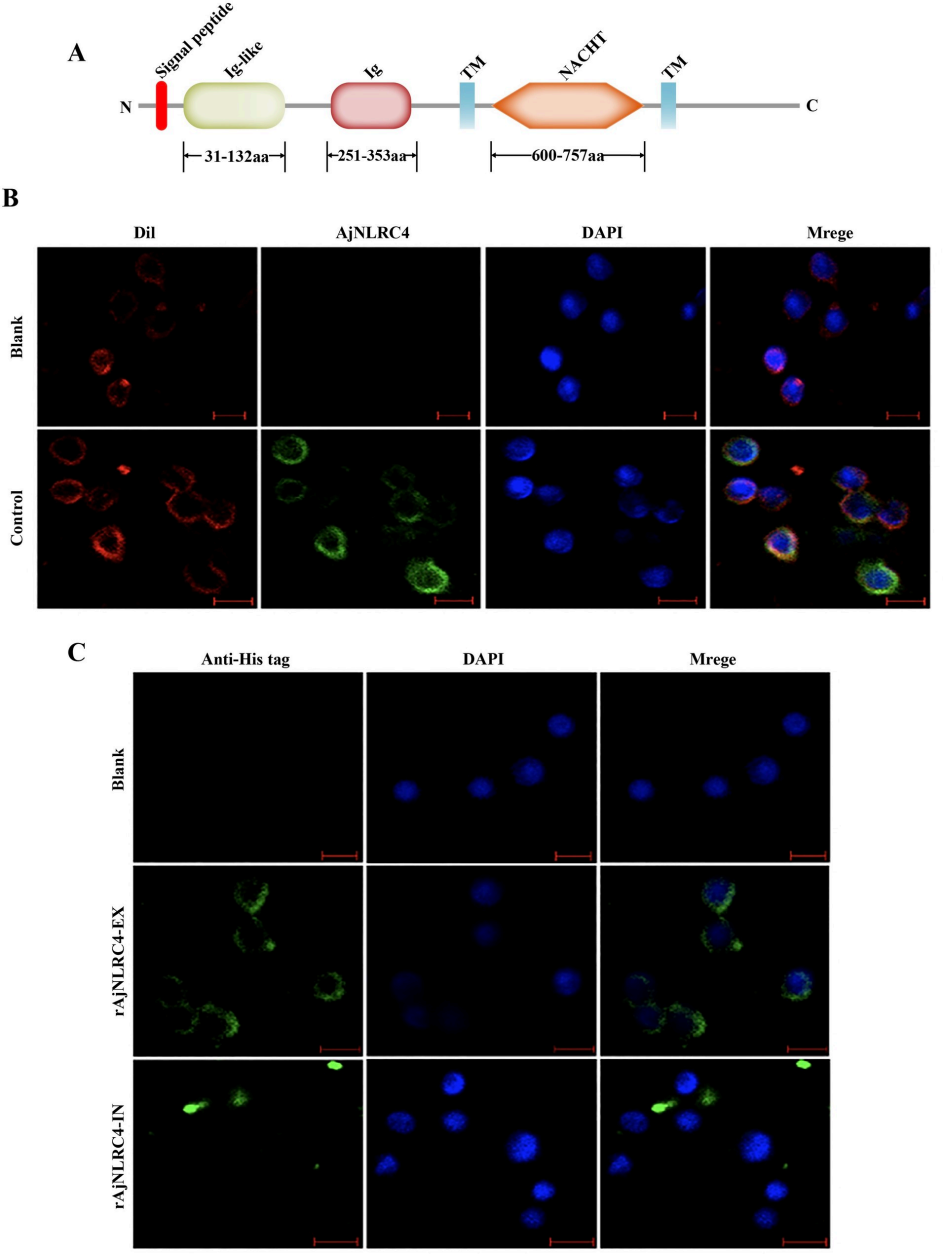
AjNLRC4 is located in the membrane of sea cucumber coelomocytes. **(A)** The domain architecture of sea cucumber AjNLRC4 predicted by SMART (http://www.smart.embl-heidelberg.de/). **(B)** Analysis of AjNLRC4 subcellular distribution in sea cucumber coelomocytes. Coelomocytes were subjected to immunofluorescence staining with the cell membrane probe Dil. AjNLRC4 is localized to the cell membrane and precisely colocalized with Dil signals. Scale bar = 5 μm. **(C)** Coelomocyte binding of rAjNLRC4-EX and rAjNLRC4-IN. rAjNLRC4-EX and rAjNLRC4-IN were injected into sea cucumbers, and coelomocytes were isolated for immunohistochemistry assays to detect binding abilities with an anti-His tag antibody (green). rAjNLRC4-EX binds to the coelomocyte surface, and rAjNLRC4-IN cannot bind to the coelomocyte surface. Scale bar = 5 μm.

We use the simple modular architecture research tool (SMART) program (http://www.smart.Emblheidelberg.de/), an online tool to analyze the domains of AjNLRC4, and the results show that AjNLRC4 has two transmembrane domains, located at 483–503 aa and 776–795 aa, with a typical transmembrane helical structure. Amino acids 1–482 are located outside the cell membrane and contain Ig-like (31–132 aa) and Ig (251–353 aa) domains. The conserved NACHT domain (600–757 aa) is located in the intracellular region (Fig 1A). To confirm the subcellular distribution of the unique domains of AjNLRC4, localization analysis of AjNLRC4 is conducted with a specific antibody. The results show that green fluorescence signals of AjNLRC4 are concentrated in the coelomocyte membrane, which can be colocalized with the membrane-specific indicator Dil (Fig 1B). Furthermore, the cell-binding abilities of rAjNLRC4-EX (Ig-like and Ig domains) and rAjNLRC4-IN (NACHT domain) are investigated. Obvious green signals are observed on the surfaces of coelomocytes injected with recombinant AjNLRC4-EX (Fig 1C middle panel), but no signals are observed on the surfaces of coelomocytes injected with recombinant rAjNLRC4-IN (Fig 1C lower panel).

### The Ig domain of AjNLRC4 performed immune recognition functions and exhibited bacterial agglutination activities in Ca^2+^-dependent manners

**Fig 2:**
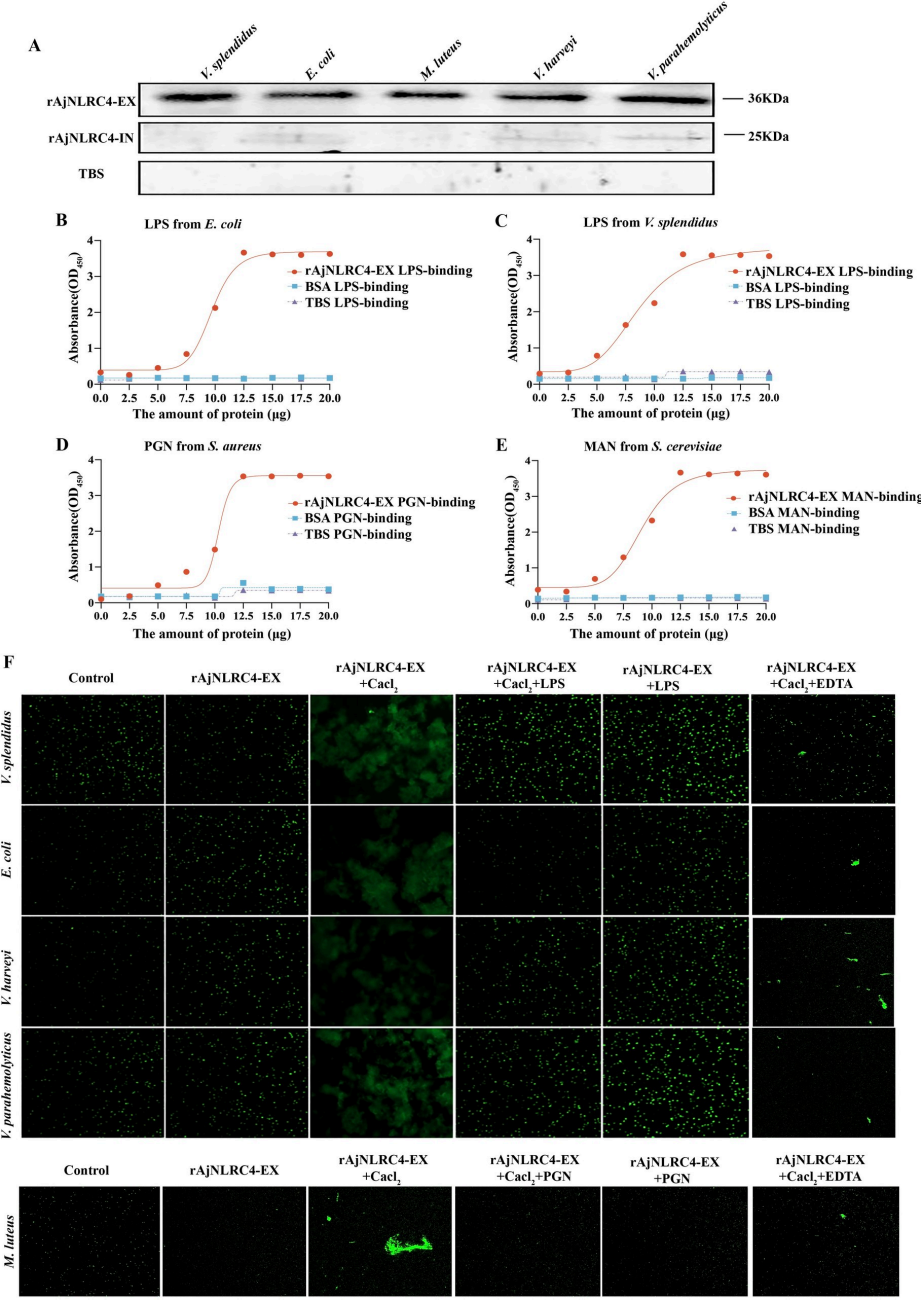
The Ig domain of AjNLRC4 performs an immune recognition function. **(A)** The bacterial binding activities of rAjNLRC4-EX and rAjNLRC4-IN were analyzed by using western blotting. Different bacteria were incubated with rAjNLRC4-EX and rAjNLRC4-IN, washed with PBS four times and then analyzed by western blotting with an anti-His antibody. TBS instead of rAjNLRC4-EX and rAjNLRC4-IN was used as the negative control. **(B-E)** The binding activities of rAjNLRC4-EX to different polysaccharides were analyzed with ELISA. Four polysaccharides were used for ELISA analysis (n = 5), including LPS from *E*. *coli* or *V*. *splendidus*, PGN and MAN. **(F)** rAjNLRC4-EX agglutinates different microbes in the presence of Ca^2+^. FITC-labeled microbes (green) were mixed with an equal volume of rAjNLRC4-EX (1 mg/ml) in the presence or absence of 10 mM CaCl_2_ and incubated at room temperature for approximately 1 h. BSA instead of rAjNLRC4-EX was used as the negative control. After incubation, agglutination reactions were observed under a fluorescence microscope.

Ig domain-contacting proteins have been demonstrated to play an important role in pathogen recognition [52]. Therefore, we analyze the potential abilities of both rAjNLRC4-EX and rAjNLRC4-IN to bind to bacteria *in vitro*. Western blotting assays reveal that rAjNLRC4-EX could significantly bind to all of the tested microbial strains, including G^+^ bacteria (*Micrococcus luteus*) and G^-^ bacteria (*Vibrio harvey*, *Vibrio parahemolyticus*, *Vibrio splendidus* and *Escherichia coli*) (Fig 2A upper panel). In contrast, rAjNLRC4-IN hardly binds to any microbial strain (Fig 2A middle panel). To elucidate whether the microbial binding ability of rAjNLRC4-EX is mediated by cell surface polysaccharides, ELISAs are performed to detect the capacity of rAjNLRC4-EX to bind to LPS, PGN and Man. The results indicate that rAjNLRC4-EX could bind to all of these ligands with high affinity (Fig 2B–2E).

FITC-labeled microbes are incubated with the tested proteins to check the possible agglutination activity. Immunofluorescence microscopy analysis demonstrates the strong agglutinating activities of rAjNLRC4-EX toward G^+^ bacteria (*M*. *luteus*) andG^-^ bacteria (*V*. *harvey*, *V*. *parahemolyticus*, *V*. *splendidus and E*. *coli*) in the presence of CaCl_2._ EDTA, as a Ca^2+^ chelator, significantly inhibits the agglutination ability of rAjNLRC4-EX on five bacteria even in the presence of Ca^2+^. Consistently, rAjNLRC4-EX fails to agglutinate all of the tested bacteria in the absence of CaCl_2_. More importantly, rAjNLRC4-EX loses its ability to bind to gram-negative bacteria after the addition of LPS, even in the presence of CaCl_2_ (Fig 2F).

### *V*. *splendidus* infection could induce AjNLRC4 dimerization and internalization into the cytoplasm of coelomocytes

**Fig 3:**
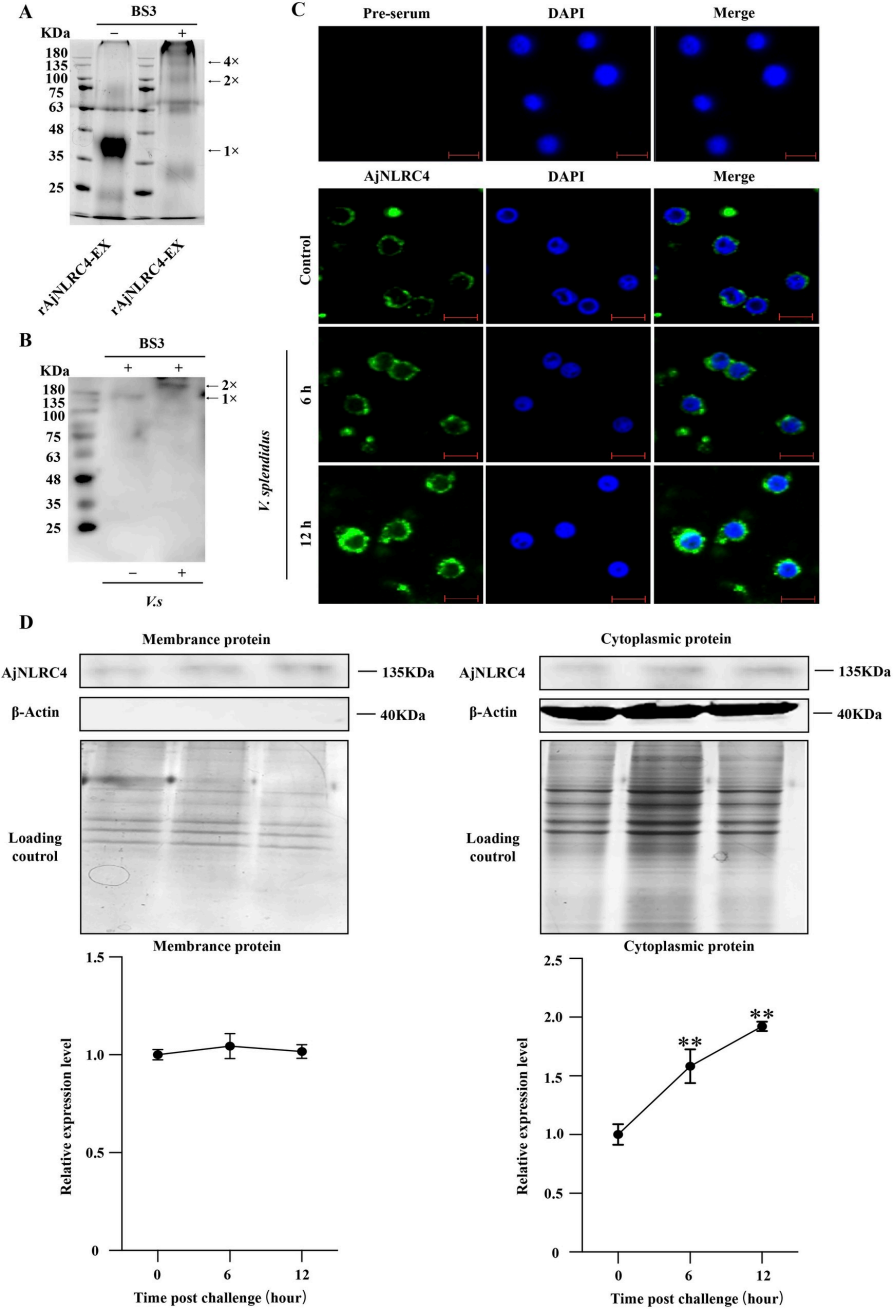
*V*. *splendidus* infection can induce AjNLRC4 dimerization and internalization into the cytoplasm of coelomocytes. **(A)** Purified rAjNLRC4-EX was analyzed using native PAGE and stained with Coomassie blue**. (B)** A dimer of AjNLRC4 was detected in coelomocytes *in vivo* using western blotting after treatment with a crosslinker (BS3). Sea cucumbers were injected with *V*. *splendidus* for 30 min, and then, coelomocytes were collected and treated with BS3. These coelomocytes were homogenized, and the extracted proteins were separated by SDS–PAGE. Western blotting was then performed using anti-AjNLRC4 antibodies. **(C)** AjNLRC4 expression in the coelomocytes of sea cucumbers was detected at 0 (untreated), 6, and 12 h postinfection with *V*. *splendidus*. Scale bar = 5 μm. **(D)** Sea cucumbers were challenged with *V*. *splendidus*, and then, the membrane and cytoplasm proteins were extracted from the coelomocytes. AjNLRC4 expression in the membrane and cytoplasm of coelomocytes was analyzed using western blotting at 0, 6, and 12 h postinfection with *V*. *splendidus*. The lower panels show the statistical analysis of three independent experiments. ***p < 0.001.

In animals, NLR oligomerization is a key step in NLR activation [27]. To analyze how AjNLRC4 responds to *V*. *splendidus* infection, AjNLRC4 oligomerization and subcellular localization are detected. Native PAGE analysis show that rAjNLRC4-EX forms different oligomers *in vitro* by molecular mass analysis (Fig 3A). To confirm that native AjNLRC4 can also be oligomerized after *V*. *splendidus* infection, the total protein from coelomocytes is analyzed by western blot with an AjNLRC4 antibody. A special band is detected in the *V*. *splendidus* group, which is two times larger in molecular weight than that in the control group (Fig 3B). We perform immunocytochemistry to detect the subcellular localization of AjNLRC4 using anti-AjNLRC4 antibodies. Under normal conditions, AjNLRC4 is mainly located on the cell membrane (Fig 3C control group). However, after *V*. *splendidus* infection for 6 to 12 h, AjNLRC4 is partially internalized into the cytoplasm (Fig 3C middle and lower panel). Proteins from the cytomembrane and cytoplasm of coelomocytes is extracted and further analyze using western blotting. The results show that AjNLRC4 is mainly detected in the membrane in the control group, and it is also partially distributed in the cytoplasm. The amount of AjNLRC4 in the cytoplasm gradually increases with *V*. *splendidus* infection (Fig 3D).

### AjNLRC4 acts as a receptor of *V*. *splendidus* and mediates its endocytosis

**Fig 4:**
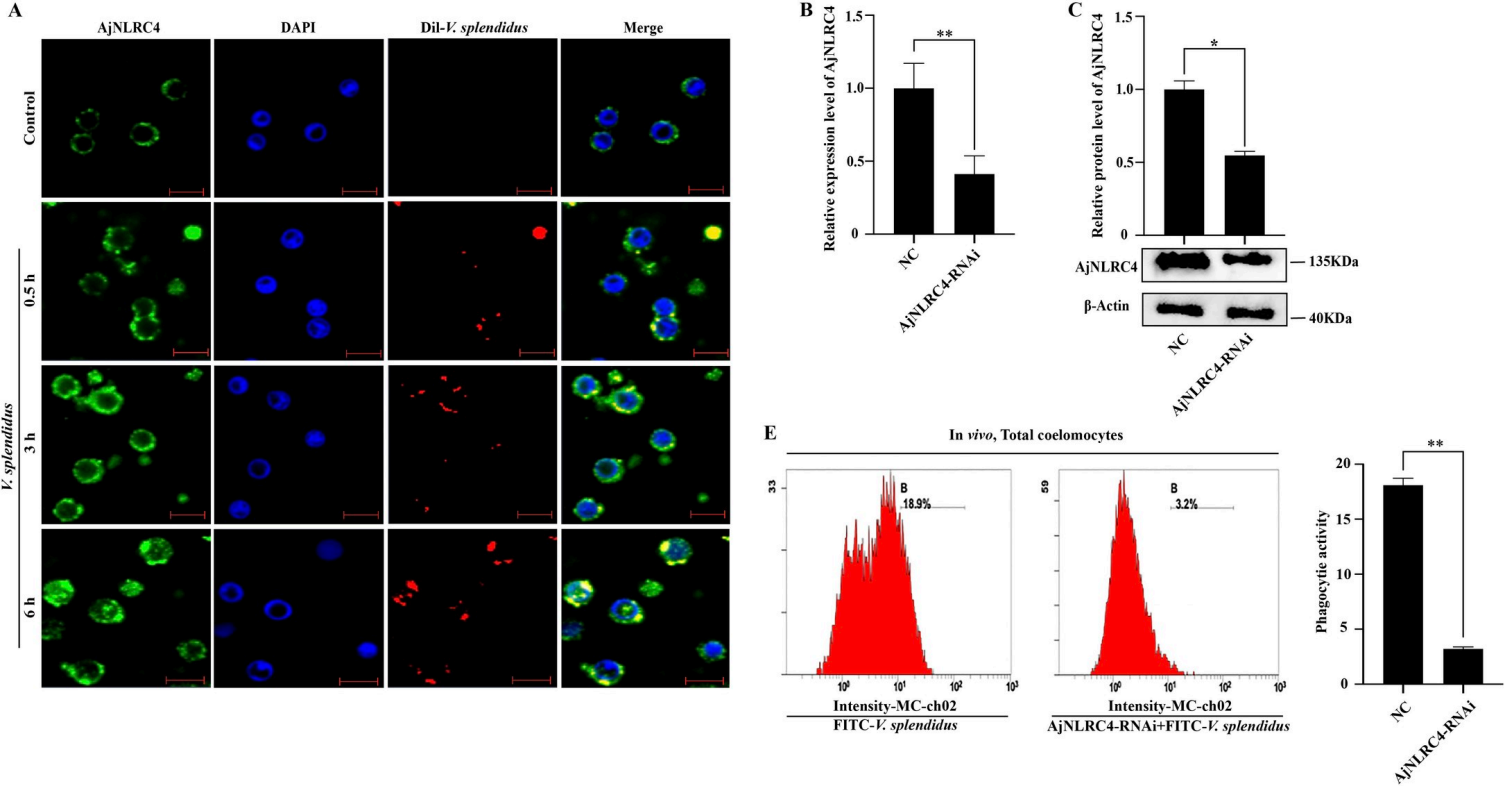
AjNLRC4 acts as a receptor of *V*. *splendidus* and mediates its endocytosis. **(A)** Immunocytochemistry was used to detect the colocalization of AjNLRC4 and Dil-labeled *V*. *splendidus* in coelomocytes. The coelomocytes were collected at different time points (0.5, 3, and 6 h) post-*V*. *splendidus* injection. Scale bar = 5 μm. The graphs are representative of three independent assays, and the proportions were calculated from those three assays, *p < 0.05. **(B-C)** The efficiency of AjNLRC4-RNAi in coelomocytes was determined using qPCR and western blotting analysis. The graphs are representative of three independent assays, and the proportions were calculated from those three assays, *p < 0.05, **p < 0.01. **(D)** Flow cytometry was performed after knockdown of AjNLRC4 to investigate the phagocytic activity of coelomocytes against *V*. *splendidus*. The graphs are representative of three independent assays, and the phagocytic activity was calculated from those three independent assays, **p < 0.01.

To determine whether the internalization of AjNLRC4 is related to the endocytosis of *V*. *splendidus*, an immunocytochemical assay is performed to detect the colocalization of AjNLRC4 and *V*. *splendidus*. We observe that AjNLRC4 colocalized with Dil-labeled *V*. *splendidus*, and this colocalization is accompanied by the cellular uptake of *V*. *splendidus* at different time points (Fig 4A). To minimize the difference in the number of phagocytic cells at different time points during *V*. *splendidus* infection, the phagocytic activity of coelomocytes is assayed before and after treatment with the cell proliferation inhibitor hydroxyurea. The results show that treatment with the cell proliferation inhibitor hydroxyurea does not affect the phagocytic activity of coelomocytes against *V*. *splendidus* (S1 Fig). Afterward, the phagocytic activity of coelomocytes against *V*. *splendidus* is further investigated, and the results showed that approximately 18.9% of coelomocytes could phagocytose *V*. *splendidus*. Knockdown of AjNLRC4 expression by siRNA transfection (Fig 4B and 4C) also significantly decreases phagocytic activity (Fig 4D).

### *V*. *splendidus* enters sea cucumber coelomocytes through multiple endocytic pathways

**Fig 5:**
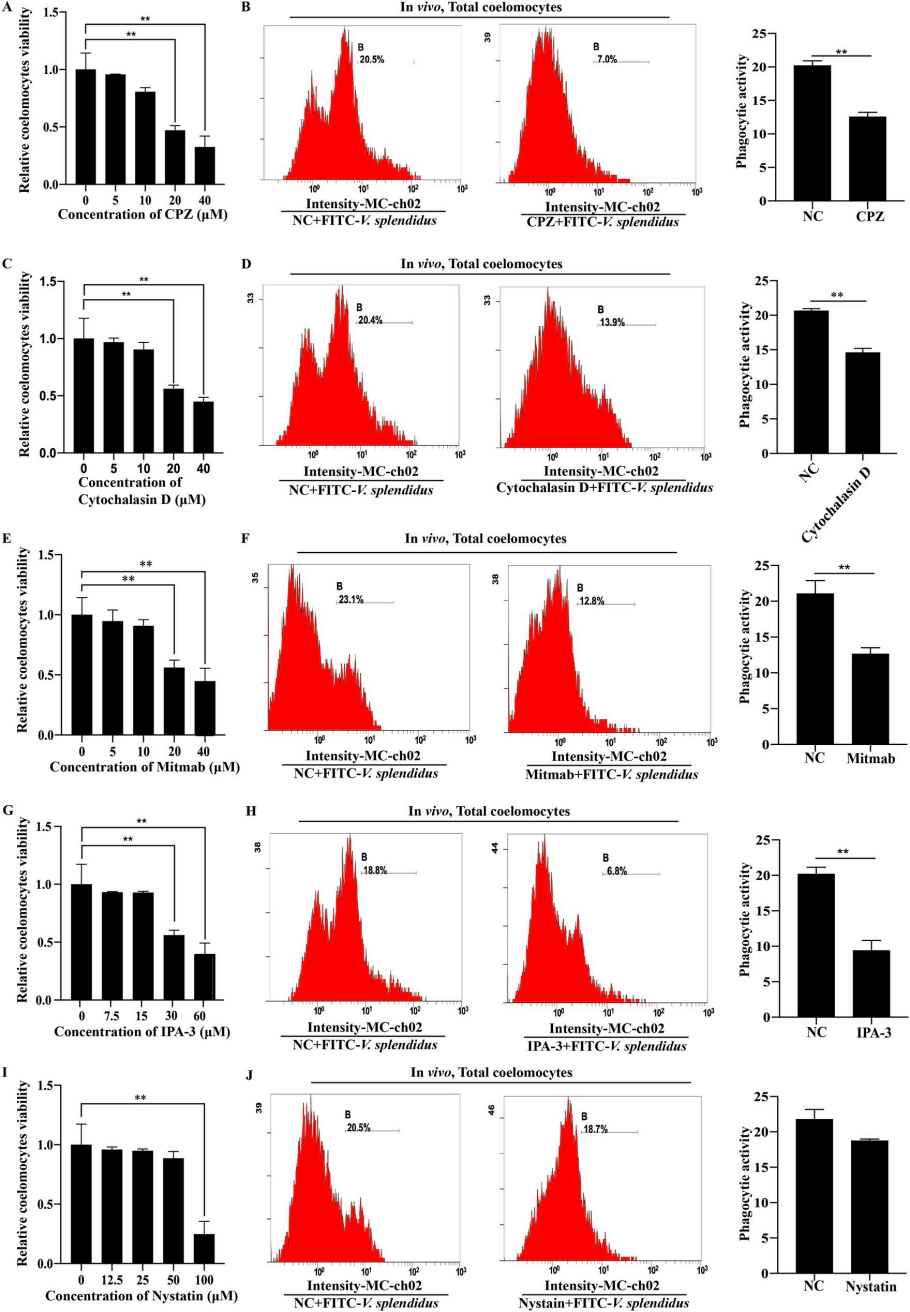
*V*. *splendidus* enters coelomocytes of sea cucumber through multiple endocytic pathways. **(A)** The effect of chlorpromazine (CPZ) on the cell viability of sea cucumbers. Sea cucumber coelomocytes were treated with increasing concentrations of CPZ for 3 h, and cell viability was calculated. **(B)** Flow cytometry was performed after CPZ inhibitor treatment to investigate the phagocytic activity of *V*. *splendidu*s. Sea cucumbers were treated with 10 μM CPZ for 3 h and then infected with FITC-labeled *V*. *splendidus* for 3 h. Flow cytometry was used to investigate the phagocytic activity of *V*. *splendidu*s. The graphs are representative of three independent assays, and the proportions were calculated from those three assays, **p < 0.01. **(C)** The effect of cytochalasin D on the cell viability of sea cucumbers. **(D)** Flow cytometry was performed after cytochalasin D inhibitor treatment to investigate the phagocytic activity of *V*. *splendidu*s. The graphs are representative of three independent assays, and the proportions were calculated from those three assays, **p < 0.01. **(E)** The effect of Mitmab on the cell viability of sea cucumbers. **(F)** Flow cytometry was performed after mitmab inhibitor treatment to investigate the phagocytic activity of *V*. *splendidu*s. The graphs are representative of three independent assays, and the proportions were calculated from those three assays, **p < 0.01. **(G)** The effect of IPA-3 on the cell viability of sea cucumbers. **(H)** Flow cytometry was performed after IPA-3 inhibitor treatment to investigate the phagocytic activity of *V*. *splendidu*s. The graphs are representative of three independent assays, and the proportions were calculated from those three assays, **p < 0.01. **(I)** The effect of nystatin on the cell viability of sea cucumbers. **(J)** Flow cytometry was performed after nystatin inhibitor treatment to test the phagocytic activity of *V*. *splendidu*s. The graphs are representative of three independent assays, and the proportions were calculated from those three assays.

To examine the pathways by which *V*. *splendidus* enters coelomocytes, we treat sea cucumber coelomocytes with five specific inhibitors of endocytic pathways to investigate the endocytic pathways on which *V*. *splendidus* relies. The results show that low doses of CPZ, an effective inhibitor of clathrin-mediated endocytosis (< 20 μM per sea cucumber), do not reduce sea cucumber viability (Fig 5A). Subsequently, sea cucumbers were treated with 10 μM CPZ and then infected with *V*. *splendidus* for 3 h. Phagocytic activity is detected by flow cytometry, and phagocytic activity is significantly decreased compared with that of the control group (Fig 5B). The protein levels of transferrin, an endosomal marker of clathrin-dependent endocytosis, are significantly downregulated after CPZ inhibitor treatment (S2A Fig). Similarly, treatment with 10 μM cytochalasin D, 10 μM mitmab and 15 μM IPA-3 inhibit *V*. *splendidus* phagocytosis (Fig 5C–5H), and the corresponding markers F-Actin/G-Actin, Rab5, and dynamin are all induced (S2B–S2D Fig). In contrast, treatment with 50 μM nystatin (an inhibitor of caveolin-mediated endocytosis) does not change the phagocytic activity of coelomocytes (Fig 5I and 5J). Caveolin 1, as an endosomal marker of caveolin-mediated endocytosis, was significantly decreased after nystatin inhibitor treatment (S2E Fig). All of these results indicate that *V*. *splendidus* phagocytosis was dependent on the clathrin-, macropinocytosis-, actin- and dynamin-mediated phagocytic pathways but not on the caveolin-mediated endocytic pathway.

### Internalized AjNLRC4 promoted *V*. *splendidus* phagocytosis via the actin-mediated endocytic pathway

**Fig 6:**
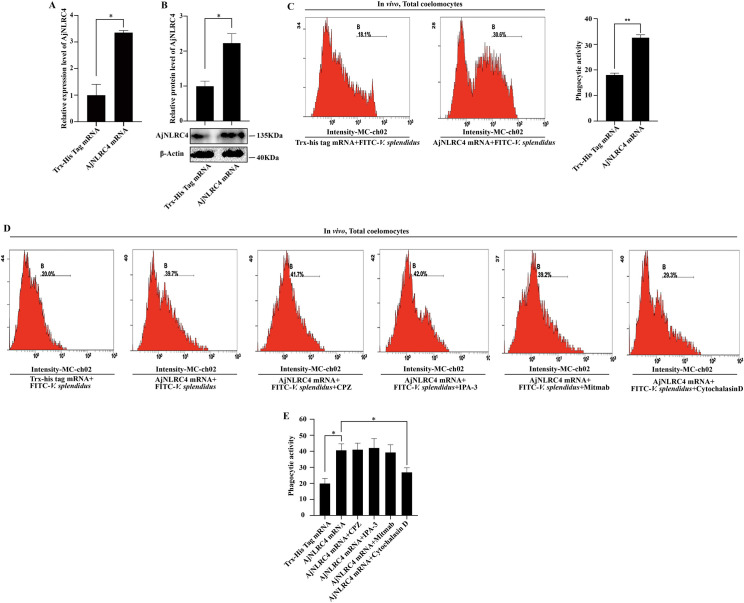
Internalized AjNLRC4 promotes *V*. *splendidus* phagocytosis via the actin-mediated endocytic pathway. **(A-B)** The efficiency of AjNLRC4 overexpression in coelomocytes, as determined using qPCR and western blotting analysis. The graphs are representative of three independent assays, and the proportions were calculated from those three assays, *p < 0.05. **(C)** Flow cytometry was performed to investigate the phagocytic activity of *V*. *splendidus* after the overexpression of AjNLRC4. The graphs are representative of three independent assays, and the proportions were calculated from those three assays, **p < 0.01. **(D-E)** After the overexpression of AjNLRC4, the inhibitors were added, and then, flow cytometry was performed to detect the phagocytic activity.

*V*. *splendidus* could enter coelomocytes through clathrin-, micropinocytosis-, actin- and dynamin-dependent pathways, but which endocytic pathway is required for the AjNLRC4-mediated phagocytosis of *V*. *splendidus* is not known. To investigate which endocytic pathway is required for the AjNLRC4-mediated phagocytosis of *V*. *splendidus*, AjNLRC4 mRNA is overexpressed by mRNA injection (Fig 6A and 6B). Under this condition, coelomocyte phagocytic activity is significantly increased (Fig 6C), and only cytochalasin D blocks AjNLRC4-mediated phagocytic activity by four different endocytic pathway inhibitor treatments (Fig 6D and 6E). There are no significant changes in the other groups (Fig 6D and 6E).

### Actin-mediated endocytic pathway activation requires the interaction between the NACHT domain and actin

**Fig 7:**
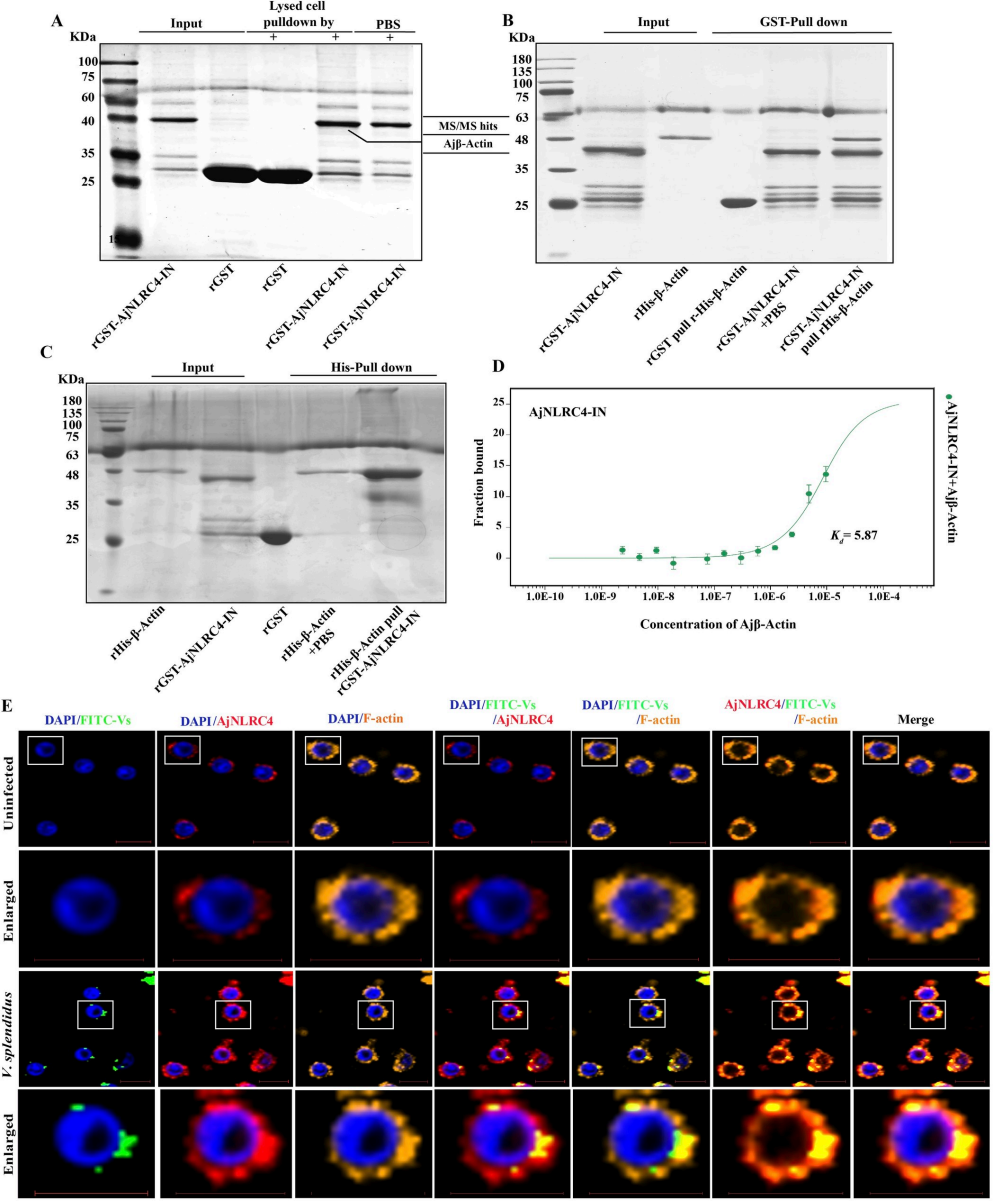
The actin-mediated endocytic pathway is activated in a NACHT domain- and actin interaction-dependent manner. **(A)** Identification of AjNLRC4-NACHT interacting proteins from host cells. Coelomocyte homogenates (20 mL) were incubated with 20 mg rGST-AjNLRC4-NACHT or rGST tag with rotation at 4°C for 3 h. The mixture was then passed through a GST-resin column. The bound proteins were eluted and analyzed by SDS–PAGE. The bands of interest were excised and analyzed by MS/MS. **(B-C)** Interactions between His-tagged β-actin and GST-tagged AjNLRC4-NACHT were detected using pull-down assays. **(D)** Microscale thermophoresis (MST) assays of the interaction between GST-AjNLRC4-IN and His-β-Actin. The recombinant proteins were contained in NT standard capillaries. The solid curve is the fit of the data points to the standard *K*_*d*_-fit function. The experiment was repeated at least three times. **(E)** Immunocytochemistry was used to detect the colocalization of AjNLRC4, F-actin and FITC-labeled *V*. *splendidus* in coelomocytes. Scale bar = 5 μm.

To explore the mechanism that underlies AjNLRC4-mediated actin-dependent endocytosis, proteins that potentially interact with AjNLRC4-NACHT are identified by pull-down assays and MS/MS analysis. Unexpectedly, among the analyzed proteins, β-actin (BAH79732) is identified as a possible AjNLRC4-IN interacting protein (Fig 7A). Furthermore, reverse GST and His pull-down assays also confirm this interaction (Fig 7B and 7C). To further verify the binding kinetics between AjNLRC4-IN and Ajβ-actin, MST assays are employed, and the dissociation constant (*K*_*d*_) of AjNLRC4-IN and Ajβ-actin is 5.87 μM (Fig 7D). All of these results suggest that AjNLRC4 most likely promotes *V*. *splendidus* entry by interacting with the cytoskeleton of sea cucumber coelomocytes. Subsequently, the colocalization of AjNLRC4, F-actin and FITC-labeled *V*. *splendidus* is investigated, and it is found that AjNLRC4 could colocalize with F-Actin. During infection with *V*. *splendidus*, the colocalization of AjNLRC4, F-actin and FITC-labeled *V*. *splendidus* is also observed (Fig 7E). The colocalization of AjNLRC4, F-actin and FITC-labeled *V*. *splendidus* indicates that AjNLRC4 promotes *V*. *splendidus* entry by interacting with the actin cytoskeleton of sea cucumber coelomocytes.

### AjNLRC4 mediates the endocytosis of *V*. *splendidus* by regulating the Arp2/3 complex to mediate actin polymerization and cytoskeletal rearrangement

**Fig 8:**
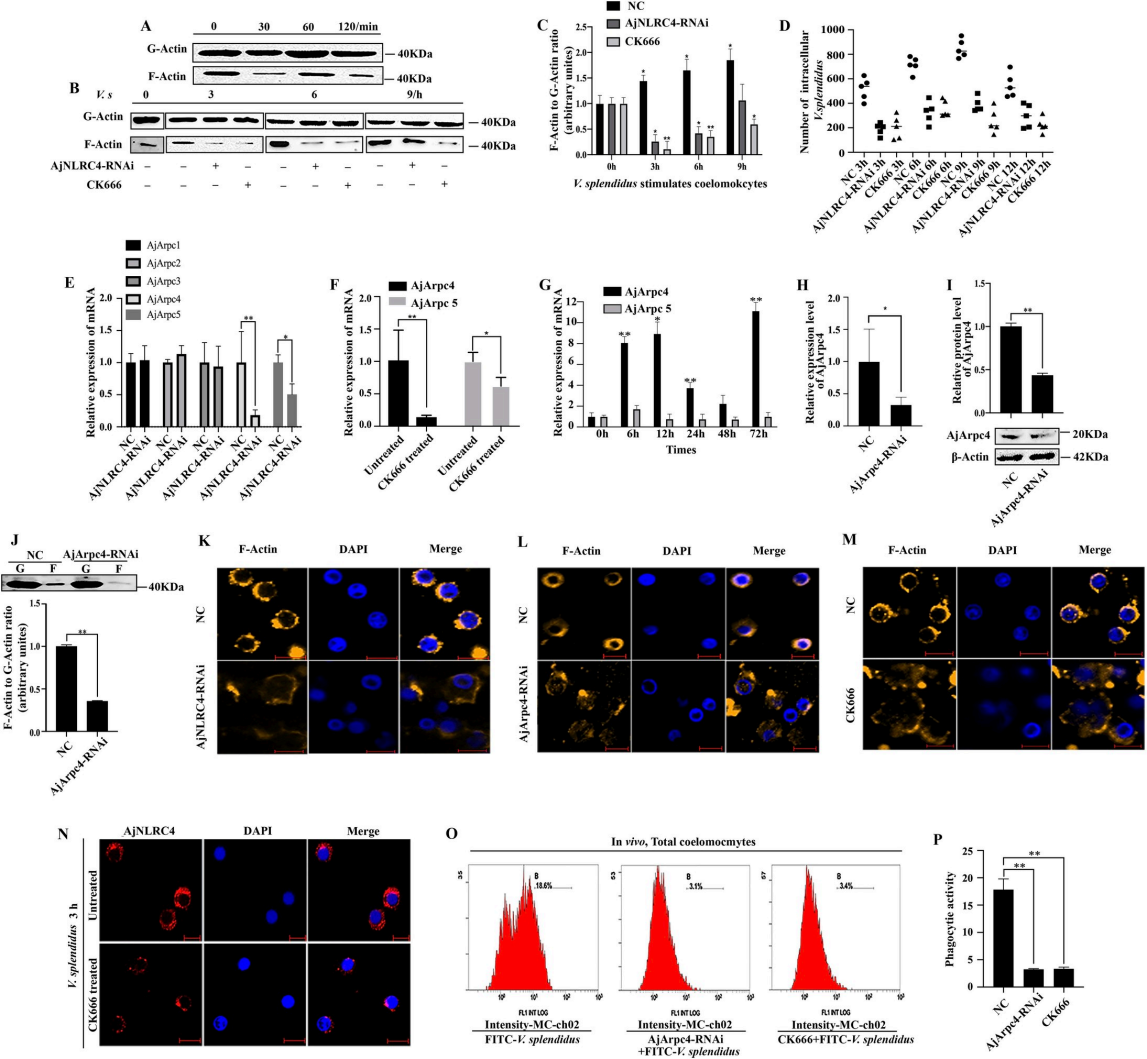
AjNLRC4 mediates the endocytosis of *V*. *splendidus* by regulating the Arpc2/3 complex to mediate actin polymerization and cytoskeletal rearrangement. **(A)** G-actin and F-actin were extracted from the coelomocytes of healthy sea cucumbers. (**B-C)** The change in the ratio F-actin/G-actin after treatment with AjNLRC4-RNAi or the cytoskeletal nucleation inhibitor CK666 was detected by western blot. **(D)** Quantification of the number of *V*. *splendidus* cells internalized into coelomocytes. After *V*. *splendidus* was coincubated with coelomocytes, the unattached bacterial cells were washed away. The numbers of cells associated with and/or internalized into coelomocytes were counted on solid plates after the detachment of the coelomocytes with trypsin-like enzyme and the lysis of the coelomocytes with Triton X-100. The *V*. *splendidus* cells internalized into the coelomocytes were counted after killing the external *V*. *splendidus* with gentamicin, detaching the coelomocytes with trypsin-like enzyme and lysing the coelomocytes with Triton X-100. The horizontal bars represent the medians. The *p* values were calculated by the t test for paired samples, and *p <0.05 indicated significant differences. **(E)** AjArpc2/3/4/5 mRNA expression levels were measured after treatment with AjNLRC4-RNAi. The graphs are representative of three independent assays, and the proportions were calculated from those three assays, *p < 0.05, **p < 0.01. **(F)** AjArpc4/5 mRNA expression levels were measured after CK666 treatment. The graphs are representative of three independent assays, and the proportions were calculated from those three assays, *p < 0.05, **p < 0.01. **(G)**, Analysis of AjArpc4/5 expression in *V*. *splendidu*s-infected sea cucumbers. The vertical bars represent the mean ± S.D. (N = 3). **(H-I)** The efficiency of AjArpc4-RNAi in coelomocytes was determined using qPCR and western blotting analysis. The graphs are representative of three independent assays, and the proportions were calculated from those three assays, *p < 0.05. **(J)** western blotting revealed the change in the ratio F-actin/G-actin after treatment with AjArpc4-RNAi. The graphs are representative of three independent assays, and the proportions were calculated from those three assays, **p < 0.01. **(K-M)** Immunocytochemistry was performed after knockdown of AjNLRC4 or AjArpc4 or treatment with CK666 (actin polymerization inhibitor) to investigate the depolymerization of actin cytoskeleton microfilaments. **(N),** Sea cucumbers were injected with CK666 inhibitor for 24 hours and then injected with *V. splendidus*. After 3 h, coelomocytes were collected from the sea cucumbers, and immunocytochemistry was performed to analyze the interactions related to the internalization of AjNLRC4 using AjNLRC4 antisera as the primary antibody. The secondary antibody was labeled with Cy3 (red). The cell nuclei were stained with DAPI (blue), and then, the cells were observed under a laser scanning confocal microscope. Scale bar = 5 μm. **(O-P),** Flow cytometry was performed to investigate the phagocytic activity of *V*. *splendidu*s after AjArpc4-RNAi or CK666 inhibitor treatment. The graphs are representative of three independent assays, and the proportions were calculated from those three assays, **p < 0.01.

To explore the role of the cytoskeleton in the process of *V*. *splendidus* infection, the cytoskeletal components G-actin and F-actin are isolated, and changes in depolymerization/polymerization are analyzed after *V*. *splendidus* infection. The results show that the G-actin and F-actin components of coelomocytes are successfully separated (Fig 8A); as the duration of *V*. *splendidus* infection increased, the content of F-actin increases, which indicates that the cytoskeleton exhibits an aggregated rearrangement (Fig 8B and 8C). This result indicates that the actin cytoskeleton plays an important role in the process of *V*. *splendidus* infection. As the time that *V*. *splendidus* infection increases, *V*. *splendidus* could be internalized into coelomocytes, replicated to 9 h post infection, and then be eliminated by the host immune system (Fig 8D). Knockdown of AjNLRC4 or treatment with an actin polymerization inhibitor (CK666) result in a significant decrease in the F-actin content (Fig 8B and 8C). Moreover, internalization experiments prove that the number of *V*. *splendidus* cells in coelomocytes is also significantly reduced (Fig 8D). To further analyze the mechanism by which AjNLRC4 regulates the cytoskeleton, we knock down AjNLRC4 expression and observe the polymerization of F-actin. Knockdown of AjNLRC4 expression depolymerizes F-actin, which inhibits the maintenance of its original morphology (Fig 8K). In addition, CK666 is also used to treat coelomocytes. In the treated coelomocytes, F-actin is also depolymerized (Fig 8M), and the activity of *V*. *splendidus* phagocytosis is also significantly reduced (Fig 8O and 8P). To analyze the interactions related to the internalization of AjNLRC4, coelomocytes are treated with CK666, and the subcellular localization of AjNLRC4 in these coelomocytes is detected. The internalization of AjNLRC4 is significantly inhibited after treatment with the CK666 inhibitor (Fig 8N).

The Arp2/3 complex plays an indispensable role in inducing actin polymerization and cytoskeletal rearrangement. To explore the mechanism by which AjNLRC4 affects the polymerization of actin, we further investigate the effect of AjNLRC4 knockdown on the Arp2/3 complex. We identify five subunits of the Arpc2/3 complex in sea cucumbers, namely, Arpc1a, Arpc2, Arpc3, Arpc4, and Arpc5. After knocking down AjNLRC4 expression or treatment with the CK666 inhibitor, the expression of Arpc4 and Arpc5 is significantly downregulated, and the expression of other Arpc2/3 complex subunits remains unchanged (Fig 8E and 8F). Then, we investigated the expression of Arpc4 and Arpc5 during infection with *V*. *splendidus*. Only the expression of Arpc4 is significantly upregulated, and the expression of Arpc5 does not change (Fig 8G). These results suggest that Arpc4 responds to the stress of *V*. *splendidus* infection. Additionally, after knocking down Arpc4, the levels of F-actin/G-actin are significantly decreased (Fig 8J), and F-actin was depolymerized, which inhibits the maintenance of its original morphology (Fig 8K). Moreover, phagocytic activity is also significantly reduced (Fig 8N). Taken together, these results suggest that AjNLRC4 mediates the endocytosis of *V*. *splendidus* by regulating the Arpc2/3 complex to mediate actin polymerization and cytoskeletal rearrangement.

### Endocytosed *V*. *splendidus* is further cleared via lysosome degradation

**Fig 9:**
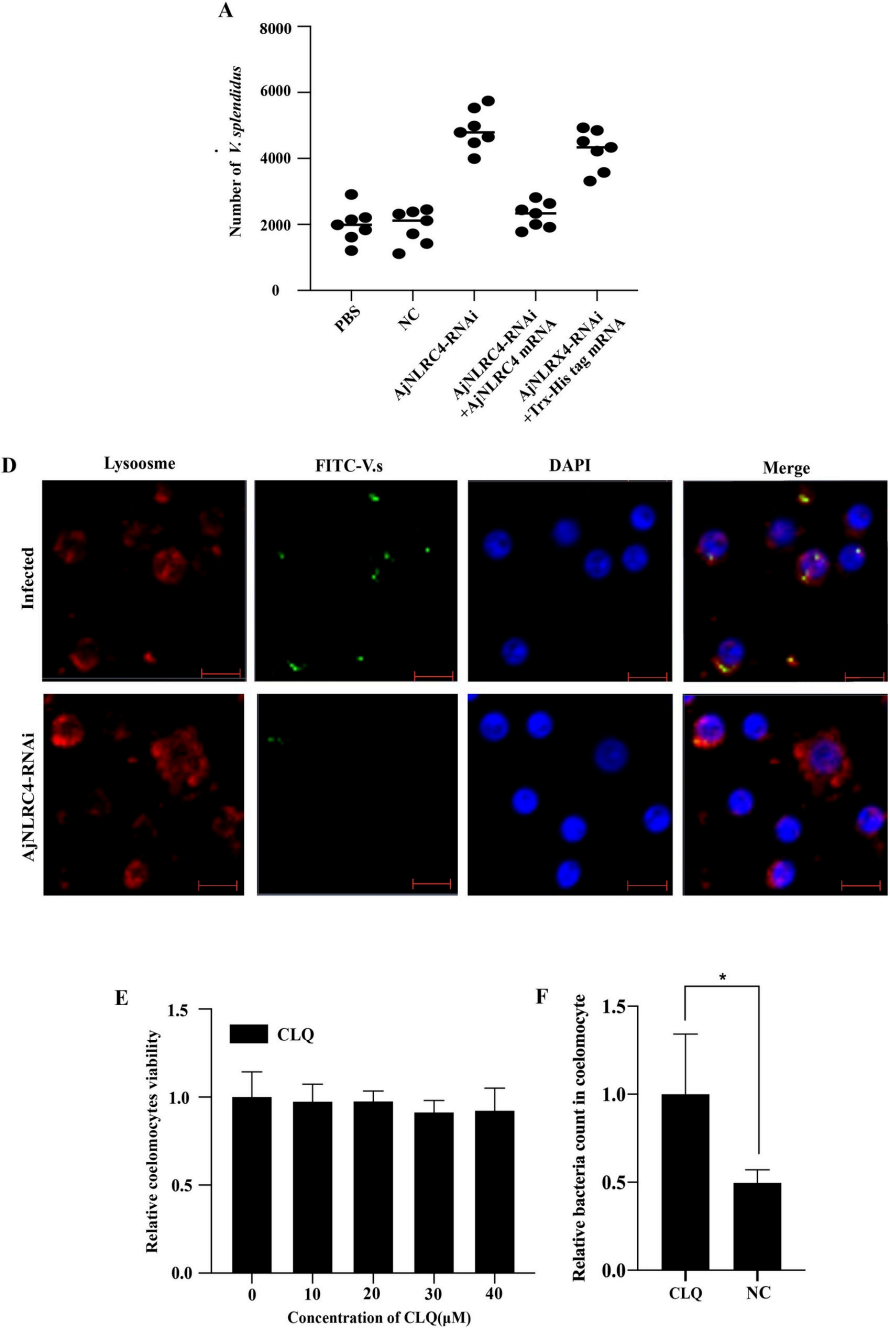
Endocytosed *V*. *splendidus* is further cleared via lysosome degradation. **(A)** Analysis of the *V*. *splendidus* clearance ability of AjNLRC4. Each dot represents a group. The horizontal bars represent the medians. **(B)** Colocalization of ingested *V*. *splendidus* and lysosomes. Twenty-four hours after the injection of 40 μM CLQ, FITC-labeled *V*. *splendidus* was injected into sea cucumbers. After another 3 h, coelomocytes were collected, incubated with LysoBrite Red to label the lysosomes, stained with DAPI and then observed under a laser-scanning confocal microscope. Scale bar = 5 μm. **(C)** Effect of chloroquine (CLQ) on the cell viability of sea cucumbers. Sea cucumbers were treated with increasing concentrations of CLQ for 24 h, and the cell viability was calculated. The graphs are representative of three independent assays, and the proportions were calculated from those three assays, *p < 0.01. **(D)** The relative number of intracellular *V*. *splendidus* after CLQ injection was determined by internalization assay. The values are presented as the mean ± SD (n = 3). The asterisks indicate significant differences: *p < 0.05.

After the internalization of microorganisms, phagosomes subsequently fuse with intracellular granules to form phagolysosomes, within which microbes are killed by a combination of nonoxidative and oxidative mechanisms [53,54]. To further investigate the effect of AjNLRC4-mediated endocytosis on *V*. *splendidus*, a bacterial clearance assay is conducted after abnormal expression of AjNLRC4. The results show that the number of *V*. *splendidus* cells is significantly increased after the knockdown of AjNLRC4 expression, and the number of *V*. *splendidus* cells is markedly decreased after AjNLRC4 overexpression (Fig 9A). Furthermore, after rescuing the expression of AjNLRC4 in the RNAi group by injection of AjNLRC4 mRNA, the number of surviving *V*. *splendidus* cells is decreased compared with the number in the RNAi group. To assess whether the injected AjNLRC4 mRNA is not targeted by AjNLRC4-RNAi in the rescue experiment, the efficiency of AjNLRC4-RNAi in coelomocytes at different times is determined first by qPCR, and then, the relative expression of AjNLRC4 mRNA in the rescue experiment is also investigated. The results indicate that AjNLRC4 mRNA levels could be significantly induced at 6 h and 12 h after AjNLRC4 mRNA injection(S3 Fig).

To further study whether lysosomes are involved in the elimination of *V*. *splendidus*, the colocalization of *V*. *splendidus* and lysosomes is analyzed. The results show that the labeled *V*. *splendidus* cells colocalized with lysosomes, which were stained with LysoBrite Red, in coelomocytes (Fig 9B). After knockdown of AjNLRC4 expression, the colocalization of *V*. *splendidus* and lysosomes in coelomocytes is significantly decreased (Fig 9B). To confirm that lysosomes are involved in *V*. *splendidus* degradation, the lysosomal inhibitor chloroquine (CLQ) is injected into sea cucumbers, and the elimination of *V*. *splendidus* is assessed. Low doses (<40 μM) of CLQ do not decrease sea cucumber viability (Fig 9C). After injection of 40 μM CLQ into *V*. *splendidus*-infected sea cucumbers, the number of surviving *V*. *splendidus* cells is obviously increased compared with the number of surviving *V*. *splendidus* cells in the control group (Fig 9D), which indicates that lysosomes are involved in the elimination of *V*. *splendidus*.

## Discussion

**Fig 10:**
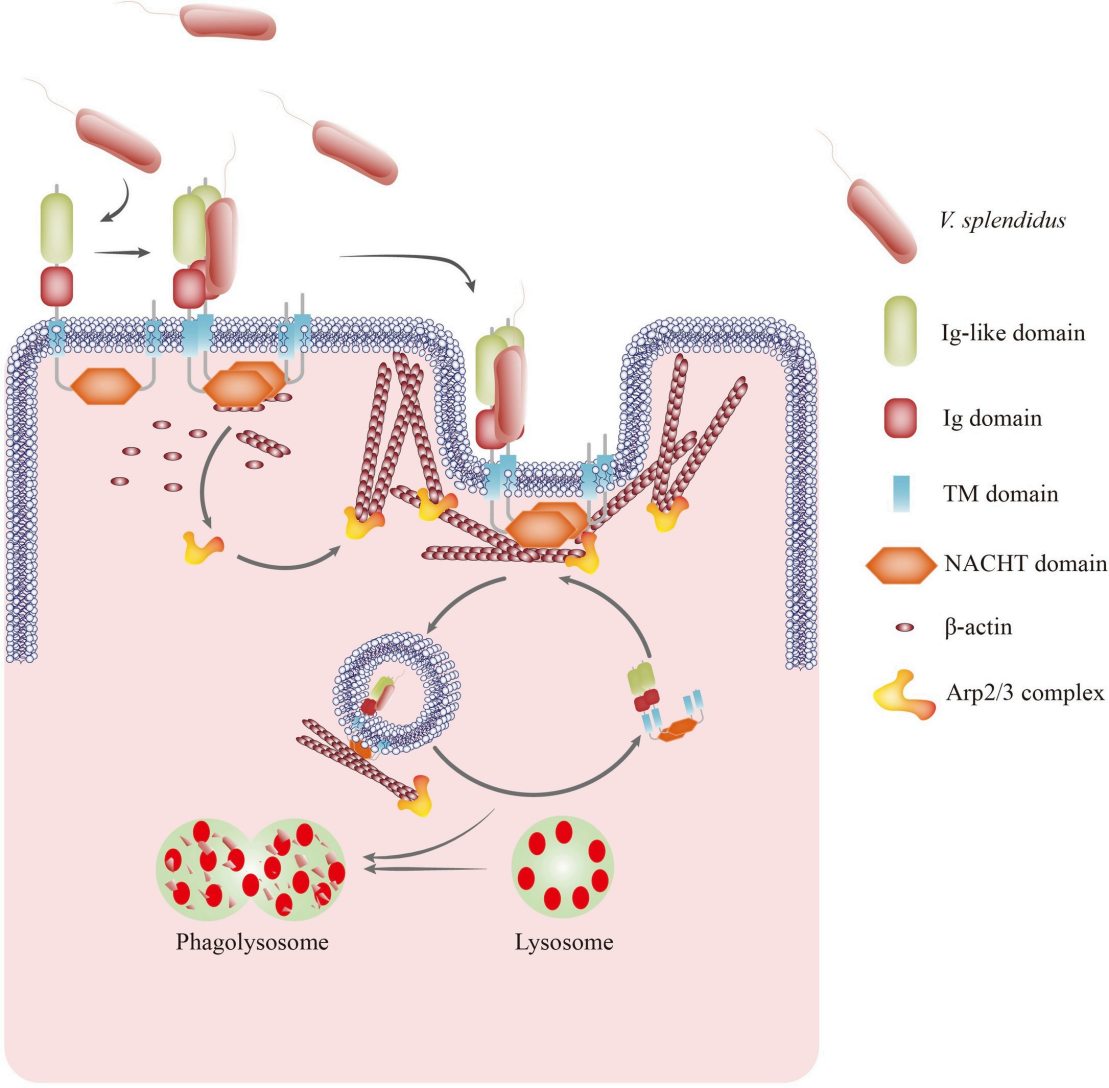
Schematic of the role of AjNLRC4 in promoting *V*. *splendidus* endocytosis as a transmembrane receptor. *V*. *splendidus* binds to the extracellular domains of AjNLRC4 and activates receptor-mediated endocytosis. AjNLRC4 oligomerizes, and the intracellular domain of AjNLRC4 interacts with Ajβ-Actin, forming a protein complex that facilitates *V*. *splendidus* internalization through the actin-mediated endocytosis pathway by Arp2/3 complex-mediated cytoskeleton polymerization and rearrangement. Finally, *V*. *splendidus* is degraded in coelomocyte phagolysosomes, which effectively restricts *V*. *splendidus* infection in sea cucumbers.

Phagocytosis is a basic process of the innate immune response and plays a key role in the ingestion and elimination of invaders. The activation of phagocytosis requires some cell membrane surface receptors, such as integrins, FcγRs, and scavenger receptors (SRs), in model animals. However, to the best of our knowledge, the role of NLRs in modulating phagocytosis is still far from well understood. In this study, we first identified an NLR with a novel structure, namely, AjNLRC4, which is located on the cell membrane of *A*. *japonicus*; AjNLRC4 is the phagocytic receptor of *V*. *splendidus* on sea cucumber coelomocytes. AjNLRC4 interacts with *V*. *splendidus* and Ajβ-Actin to promote coelomocyte phagocytosis in an actin-dependent manner. The Arp2/3 complex is regulated by AjNLRC4 to mediate actin polymerization and cytoskeletal rearrangement and ultimately leads to *V*. *splendidus* degradation in phagolysosomes (Fig 10). To our knowledge, AjNLRC4 is the first report of a novel cell membrane receptor that mediates antibacterial effects in sea cucumber.

Biological studies have shown that the number of NLR receptors expressed in a given species is markedly variable. Because orthologs of NLRs have not been found in *Drosophila* and *C*. *elegans*, it is first thought that the NLR family originated in teleost fish [55,56]. However, analysis of the sea urchin genome sequence revealed that more than 200 NLR-like genes are present in this animal [57–59]. This circumstance leads to a reconsideration of the evolutionary origins of NLRs, and it is plausible that precursors of NLRs exist in basal metazoans. In addition, NOD-containing proteins are found in the genomes of the sea anemones *Nematosella vectensis* and *Hydra magnipapillata*, which contain approximately 72 and 290 genes, respectively [60,61]. These species belong to the phylum cnidaria, whose evolutionary branch precedes the bilateria split, and they are considered to be one of the oldest metazoans on the planet. This circumstance reinforces the idea that NLRs in vertebrates share a common ancestor in basal metazoans. There is a close evolutionary relationship between sea cucumbers and these species, which also implies that there could be ancient NLRs in sea cucumbers. Interestingly, *H*. *magnipapillata* expresses a NOD protein that is similar to AjNLRC4, and this protein also lacks the C-terminal LRR domain found in higher organisms as well as the common N-terminal CARD and PYD domains and other characteristic domains. This protein contains a DEATH domain at its N-terminus and a core NACHT domain. Therefore, the deletion of the LRR and N-terminal characteristic domains is very common in early ancestors of NLRs, which also suggests that these domains could have evolved later. Therefore, we speculate that AjNLRC4 lacks LRR and CARD domains, which could indicate that the early ancestors of NLRC lacked these effector domains and later acquired LRR and CARD domains. Second, AjNLRC4 has a NACHT domain that is very similar to the NACHT domain of other NLRCs, which indicates that AjNLRC4 could have evolved from other ancient NLRC4-like molecules. NLRs bear strong similarities to plant disease resistance (R) proteins in terms of structure and function [62]. Plant R proteins are expressed in both cytoplasmic and membrane-bound forms. In plants, the NLR immune receptor Tm-22 functions at the plasma membrane (PM) and recognizes viral MP independent of its PD (plasmodesmata) localization [63]. In animals, NLRX1 contains a mitochondria-targeting sequence (MTS) [64,65], and it is the only molecule in the NLR family with an N-terminal transit peptide that can guide it to the mitochondria. The position of NLRX1 in the mitochondria might not be fixed because recent studies have found that NLRX1 localizes to both the mitochondrial outer membrane (MOM) and the mitochondrial matrix. In this study, it was found that AjNLRC4 is located directly on the cell membrane. The direct localization of AjNLRC4 on the cell membrane could be related to its N-terminal Ig domain. It has been reported that most Ig domain-containing proteins (ICPs) are transmembrane proteins, such as ICP-1 [66], JAM-A-L [34], Siglec-1 [67] and IgIT [68], which have an extracellular domain (containing one or more Ig-like domains), a TM domain, and a cytoplasmic region [69]. AjNLRC4 and ICPs are very similar in composition. AjNLRC4 is located on the cell membrane, similar to most ICPs, and its Ig domain is located outside the cell membrane. The Ig domain mediates a variety of functions, including pathogen recognition, cell adhesion, and immune system regulation [70]. In this study, the extracellular Ig domain of AjNLRC4 exhibited a strong ability to recognize and bind a variety of pathogenic microorganisms. AjNLRC4 can also agglutinate a variety of different pathogenic microorganisms through its Ig domain, similar to the ICP member hemolin [71]. Although the classic LRR recognition module is missing, the Ig domain of another classic recognition module completely replaces the LRR domain to allow AjNLRC4 to function in immune recognition. In addition, the extensive microbial ligand binding ability of the Ig domain in the extracellular region further proves that AjNLRC4 functions as a PAMP or DAMP sensor, unlike NLRP10, which participates in immune regulation and functions as an adaptor or regulatory protein due to a lack of an LRR domain [72].

After a classic animal NLR recognizes a pathogen, activated NLRs usually oligomerize, form high molecular weight complexes known as inflammasomes, and recruit caspases to stimulate immune signaling [73]. In addition to forming inflammasomes, NLRs can also activate several signaling cascades, including the MAPK and NF-kB pathways, triggering the production of proinflammatory cytokines and chemokines that subsequently recruit immune cells to the sites of microbial infection and thereby resist infection by pathogenic microorganisms [74]. In this study, we confirmed that AjNLRC4 could form homodimers via its Ig domains after *V*. *splendidus* infection, possibly because the Ig domain can recognize bacteria and polysaccharides [75], bind to other Ig-like domains with similar structures, or interact with other molecules, such as integrins and carbohydrates, through protein–protein interactions to form homopolymers [76]. After AjNLRC4 forms a dimer, it is internalized from the cell membrane into the cytoplasm and resists pathogen infection by phagocytosing pathogenic microorganisms (Fig 10). This process is a new mechanism of NLR-mediated resistance to pathogen infection. This mechanism is completely different from the traditional mechanism by which NLRs resist pathogenic infection, and this new mechanism might be the result of the combination of the N-terminal Ig domain and the NACHT domain. Some studies suggest that domain architectures are largely conserved [77]. However, a more recent study indicates that domain architecture reinvention is a more common phenomenon than previously thought [78]. NACHT is a representative of parallel evolution of innate immune receptors. The NACHT domain contains protein–protein interaction domains that contribute to signal transduction, and this functional class of proteins is called “promiscuous” because of their tendency to associate with different domains. When compared with the list of the top 215 highly promiscuous domains in eukaryotes [79], it turned out that not only the NACHT domains themselves but also the domains they associate with, such as the Death, CARD and Ig domains, are on that list. However, only a small fraction of the possible domain combinations actually exist in nature, which suggests that domain architectures are under strong evolutionary selection [80]. For NLR family proteins, where only a limited number of protein–protein interaction domains, such as Death, CARD, DED, or PYRIN domains, can appear at the amino terminus, we obtain a clue on how such selection could be executed. These four domains belong to the death domain superfamily, which has very similar structures and modes of action [81]. Reshuffling between these domains would not incur much structural conflict with the function of controlled oligomerization facilitated by the NACHT domain. In this context, it could be worthwhile to mention that the PYRIN domain, which is not found in any currently sequenced invertebrate genome, has most likely evolved from other death domain superfamily members and represents another example of domain reshuffling. It is worthwhile to note that the combination of the Ig domain and NACHT domain could represent another example of domain reshuffling. In teleost fish, a unique NLR subfamily, subfamily C, was identified, and the members of this family possess a B30.2 (PRY-SPRY) domain, which could allow these proteins to interact with different molecules than standard NLRs and thus perform some novel function [82]. The Ig domain plays an important role in mediating phagocytosis, and it could allow AjNLRC4 to act as a phagocytic receptor that mediates phagocytosis, similar to ICP molecules.

Uptake of a microbial particle usually occurs via phagocytosis, which is induced by a pathogen-receptor interaction [83]. This study showed that AjNLRC4 could be directly used as a receptor by which *V*. *splendidus* enters cells with the internalization of AjNLRC4, and this internalization is closely related to phagocytosis. The next question is how does receptor-mediated phagocytosis take place? An extracellular signal must be transferred to the cell cytoplasm by a receptor. The intracellular domains of PRRs often interact with cytoskeletal or adaptor proteins. This interaction can lead to the transmission of extracellular interaction signals inside cells, thereby mediating phagocytosis [84]. In this study, the intracellular domain of AjNLRC4 is a NACHT domain that is characteristic of NLRs. This intracellular domain can directly interact with β-actin and is specifically involved in the actin-mediated phagocytic pathway. The direct binding of the AjNLRC4-NACHT domain to actin is consistent with the function of the classic NLR-NACHT domain. In classic NLRs, the NACHT domain extensively participates in direct interactions with other proteins to mediate a variety of immune responses. Abin-1, a negative regulator of NF-kB, is an interaction partner of NLRP10 and binds to its NACHT domain, resulting in enhanced proinflammatory signaling [85]. Through its NACHT domain, NLRX directly binds to LC3, and this interaction between the NACHT domain and LC3 is essential for *Listeria monocytogenes*-induced mitochondrial autophagy [86].

Membrane recruitment after cellular exposure to pathogenic stress is a common theme for members of the NLR family. For example, the NLRC family member NOD1 is found at the plasma membrane, where it colocalizes with F-actin. This colocalization was suggested to be a prerequisite for signaling because altering actin polymerization affects NOD1 signaling [87]. Of note, the NOD1-related protein NOD2 is also regulated by the small GTPase Rac1 [88,89] and localizes to the plasma membrane at cortical F-actin structures, similar to NOD1 [87,89,90]. Cellular actin dynamics are strictly controlled by the action of nucleation factors such as Arp2/3, which bind to the sides of pre-existing filaments and promote the growth of new filaments at these sites. Together, these results indicate an intimate association of NOD1 and NOD2 signaling with the actin cytoskeleton, although the mechanistic details remain largely unknown. In this assay, after *V*. *splendidus* infection, AjNLRC4 also colocalized with F-actin, similar to NOD1 and NOD2. However, AjNLRC4 is unlike NOD1 and NOD2 in that these molecules are recruited from the cytoplasm to the plasma membrane after pathogen stimulation. Consider NOD2 as an example. Disruption of membrane ruffles by a dominant negative form of Rac1 primed NOD2-dependent NF-kB signaling. The recruitment of NOD2 to Rac-induced dynamic cytoskeletal structures could be a strategy to both repress NOD2-dependent NF-kB signaling in unstimulated cells and rapidly mobilize NOD2 during bacterial infection [89]. However, AjNLRC4 internalized *V*. *splendidus* from the cell membrane into the cytoplasm via alterations in actin polymerization and cytoskeletal rearrangement in our case (Fig 10). On the one hand, using a medium load of *V*. *splendidus* to infect the coelomocytes, the coelomocytes could degrade *V*. *splendidus* through phagocytosis. On the other hand, a high load of *V*. *splendidus* mutually caused the death of the coelomocytes, which indicates that different doses and virulence of *V*. *splendidus* could trigger different immune responses. Interestingly, studies have reported that after a medium load of *Salmonella typhimurium* was used to infect a human cell line that overexpresses NLRC4, the cells inhibited the proliferation of *Salmonella typhimurium*. If a high load of *Salmonella typhimurium* was infected after expressing NLRC4 macrophages, the macrophages underwent cell death, which indicates that different bacterial loads can mediate different functions of NLRC4 [91]. This finding also inspires us to determine whether different doses of *V*. *splendidus* can also mediate completely different functions in the form of AjNLRC4. This question is worthy of our in-depth study. Another most relevant finding is that Aj-NLRC4 is involved in phagocytosis (Fig 10), while other (vertebrate) NLRC4 proteins are active in inflammasome activation and could even act antiphagocytically as part of inflammation [92]. We speculate that this completely different method of recruitment and the pathway by which pathogen invasion is resisted could occur because AjNLRC4 lacks the traditional LRR and CARD domains, making it unable to perform the corresponding functions of traditional NLRs. The preservation of the NACHT domain allows AjNLRC4 to perform the traditional functions associated with the NLR-NACHT domain. Most importantly, AjNLRC4 has a functionally rich and diverse Ig domain, which makes AjNLRC4 different from traditional NLRs and allows it to perform novel functions.

In conclusion, AjNLRC4 was first identified as a transmembrane receptor for *V*. *splendidus* phagocytosis in *A*. *japonicus*. AjNLRC4 recognizes *V*. *splendidus* through its extracellular Ig-like and Ig domains and further promotes AjNLRC4 oligomerization. The intracellular domain of oligomerized AjNLRC4 subsequently interacts with Ajβ-Actin to facilitate *V*. *splendidus* internalization via the actin-mediated endocytosis pathway by Arp2/3 complex-mediated cytoskeleton polymerization and rearrangement. In the end, *V*. *splendidus* is degraded in in coelomocyte phagolysosomes (Fig 10).

## Supporting information

S1 Text Support information for MIQE compliance in qPCR data. (DOCX)

S1 Fig Determination of changes in phagocytic activity of coelomocytes to *V*. *splendidus* after treatment with cell proliferation inhibitors (Hydroxyurea). 200 μg Hydroxyurea solution was injected into sea cucumbers for 6 h before FITC-labeled *V*. *splendidus* injection. Same volume of PBS were served as a control. The phagocytic activity was determined by flow cytometry. The graphs are representative of three independent assays, and the proportions were calculated from those three assays.
(TIF)

S2 Fig Perform endosomal marker analysis through Western Blot to determine the inhibitory effect of specific endocytic pathway inhibitors. **(A)** The relative protein level of transferrin after CPZ inhibitor treatment. The graphs are representative of three independent assays, and the proportions were calculated from those three assays, *p < 0.05. **(B)** The ratio of G-Actin/F-Actin after cytochalasin D inhibitor treatmen. The graphs are representative of three independent assays, and the proportions were calculated from those three assays, *p < 0.05. **(C)** The relative protein level of Rab5 after Mitmab inhibitor treatment. The graphs are representative of three independent assays, and the proportions were calculated from those three assays, *p < 0.05. **(D)** The relative protein level of dynamin after IPA-3 inhibitor treatment.The graphs are representative of three independent assays, and the proportions were calculated from those three assays, *p < 0.05. **(E)** The relative protein level of caveolin1 after nystatin inhibitor treatment. The graphs are representative of three independent assays, and the proportions were calculated from those three assays, *p < 0.05.
(TIF)

S3 Fig Reliability analysis of rescue experiment. **(A)** The efficiency of AjNLRC4-RNAi in coelomocytes at different times was determined using qPCR. The graphs are representative of three independent assays, and the proportions were calculated from those three assays, *p < 0.05, **p < 0.01. **(B)** The relative expression of AjNLRC4 mRNA in the rescue experiment. The graphs are representative of three independent assays, and the proportions were calculated from those three assays, *p < 0.05.
(TIF)
