# Supplementary material for: A unique NLRC4 receptor from echinoderms mediates Vibrio phagocytosis via rearrangement of the cytoskeleton and polymerization of F-actin
Source: PLoS Pathog. 2021 Dec 13;17(12):e1010145. doi: 10.1371/journal.ppat.1010145 (PMC8699970; doi:10.1371/journal.ppat.1010145)
Supplement: S1 Text — (DOCX) [file ppat.1010145.s001.docx]

**Supplemental Information**

**Support information for MIQE compliance in qPCR data**

1. **EXPERIMENTAL DESIGN AND SAMPLES**

siRNAs specific to AjNLRC4 were designed and synthesized by GenePharma (Shanghai, China) (Table 1). Another siRNA (negative control, NC) that was not specific for any unigenes in the *A. japonicus* transcriptome served as a negative control. These siRNAs were then dissolved in RNase-free water to generate 20 μM working solutions. For RNA interference, 10 μL AjNLRC4 siRNA and an equal volume of transfection reagent were mixed with 80 μL of phosphate-buffered saline (PBS) to prepare the transfection solution. Each sea cucumber (approximately 115 g in weight) was injected with 100 μL of the transfection solution described above by tentacle injection. The control group was injected with NC siRNA under the same conditions. Similar method was used for AjArpc4 knockdown assay. At 24 h posttransfection, control and treated coelomocytes were harvested to assess the silencing efficiency. The treated and negative control groups were set up in triplicate.

1. **NUCLEIC ACID EXTRACTION**

The total RNA was extracted from the coelomocytes of *A. japonicus* by using the RNAiso plus reagent (TaKaRa).The total RNA was extracted from the coelomocytes of *A. japonicus* by using the RNAiso plus reagent (TaKaRa) and treated with the RNase-free DNase I (TaKaRa) to remove the genomic DNA according to the manufacturer’s protocol. Polysaccharides, membranes, and unlysed cells are eliminated by centrifugation (12,000 g, 10 min, 4 ̊C). At this step the supernatant is immediately treated according to the supplier protocol. Briefly, add 200 µl of chloroform, incubate at room temperature for 2 min, and centrifuge (12,000 g, 15 min, 4 ̊C). The supernatant (600 µl emented with 500 µl of 2-propanol and centrifuged (12,000 g, 10 min, 4 ̊C) after a 10-min incubation at room temperature. The RNA pellet is washed [1 ml of 70%(v/v) ethanol and centrifugation at 8000 g for 10 min at 4 ̊C, dried at room temperature for 30 min, and resuspended in 20 µl of Milli-Q (Millipore, Bedford, MA)-treated sterile water.

1. **REVERSE TRANSCRIPTION**

Genomic DNA removal reaction

| Reagent | volume of use |
| --- | --- |
| 5×gDNA Eraser Buffer | 2μL |
| gDNA Eraser | 1μL |
| Total RNA | 1μg |
| RNase Free H_2_O | Up to 10μL |

↓

42°C 2min

↓

Reverse Transcription

(TB Green qPCR method）

| Reagent | volume of use |
| --- | --- |
| The reaction solution from the previous step | 10μL |
| PrimerScript RT Enzyme MIX 1 | 1μL |
| RT Primer | 4μL |
| 5×PrimeScript Buffer 2（for Real Time） | 4μL |
| RNase Free H_2_O | 1μL |
| Total | 20μL |

↓

37°C 15min

↓

85°C 5sec

1. **qPCR TARGET INFORMATION and qPCR OLIGONUCLEOTIDES**

| **Assay ID** | **Sequence accession number** | **Oligo ID** | **Oligonucleotide sequence and modifications (5’ to 3’)** | **Amplicon length** | ***In silico***  **verification** |
| --- | --- | --- | --- | --- | --- |
| AjNLRC4 | MN607598 | Shown in table 1 | Shown in table 1 | 189bp | Yes; specific for target |
| AjArpc1 | NCBI unpublished | Shown in table 1 | Shown in table 1 | 182bp | Yes; specific for target |
| AjArpc2 | NCBI unpublished | Shown in table 1 | Shown in table 1 | 162bp | Yes; specific for target |
| AjArpc3 | NCBI unpublished | Shown in table 1 | Shown in table 1 | 168bp | Yes; specific for target |
| AjArpc4 | NCBI unpublished | Shown in table 1 | Shown in table 1 | 151bp | Yes; specific for target |
| AjArpc5 | NCBI unpublished | Shown in table 1 | Shown in table 1 | 101bp | Yes; specific for target |
| Ajβ-Actin | EU668024.1 | Shown in table 1 | Shown in table 1 | 154bp | Yes; specific for target |

1. **qPCR PROTOCOL**

The transcripts of genes were analyzed via quantitative real-time PCR (qRT-PCR) on a Applied Biosystem 7500 real-time PCR system. According to the manufacturer's protocol, total RNA were extracted with the TRIzol reagent (Takara, Japan), and cDNA was prepared using PrimeScript™ RT reagent with gDNA Eraser Kit (Takara, Japan). Amplification was conducted in a 20uL reaction volume containing 8 uL of 1:50 diluted cDNA, 0.8 uL of each primer (listed in Table 1), 10 uL of SYBR Green, and 0.4 uL of ROX (Takara, Japan). The reaction mixtures were incubated for 2 min at 95 ̊C, followed by 40 cycles of 15 s at 95 ̊C, 15 s at 60 ̊C, and 20 s at 72 ̊C followed by a melting curve. The baseline was automatically set by the software to maintain consistency. The relative expression levels were calculated using the 2^−ΔΔ^CT method with β-actin for normalization, Each PCR trial was run in triplicate parallel reactions and repeated three times. The primer efficiency was checked. A significant difference in expression relative to expression in the control group at each time point is indicated using an asterisk for *p* < 0.05 and two asterisks for *p* < 0.01.

1. **qPCR VALIDATION**

This table details the validation for each Primer used to generate the figures in this study.


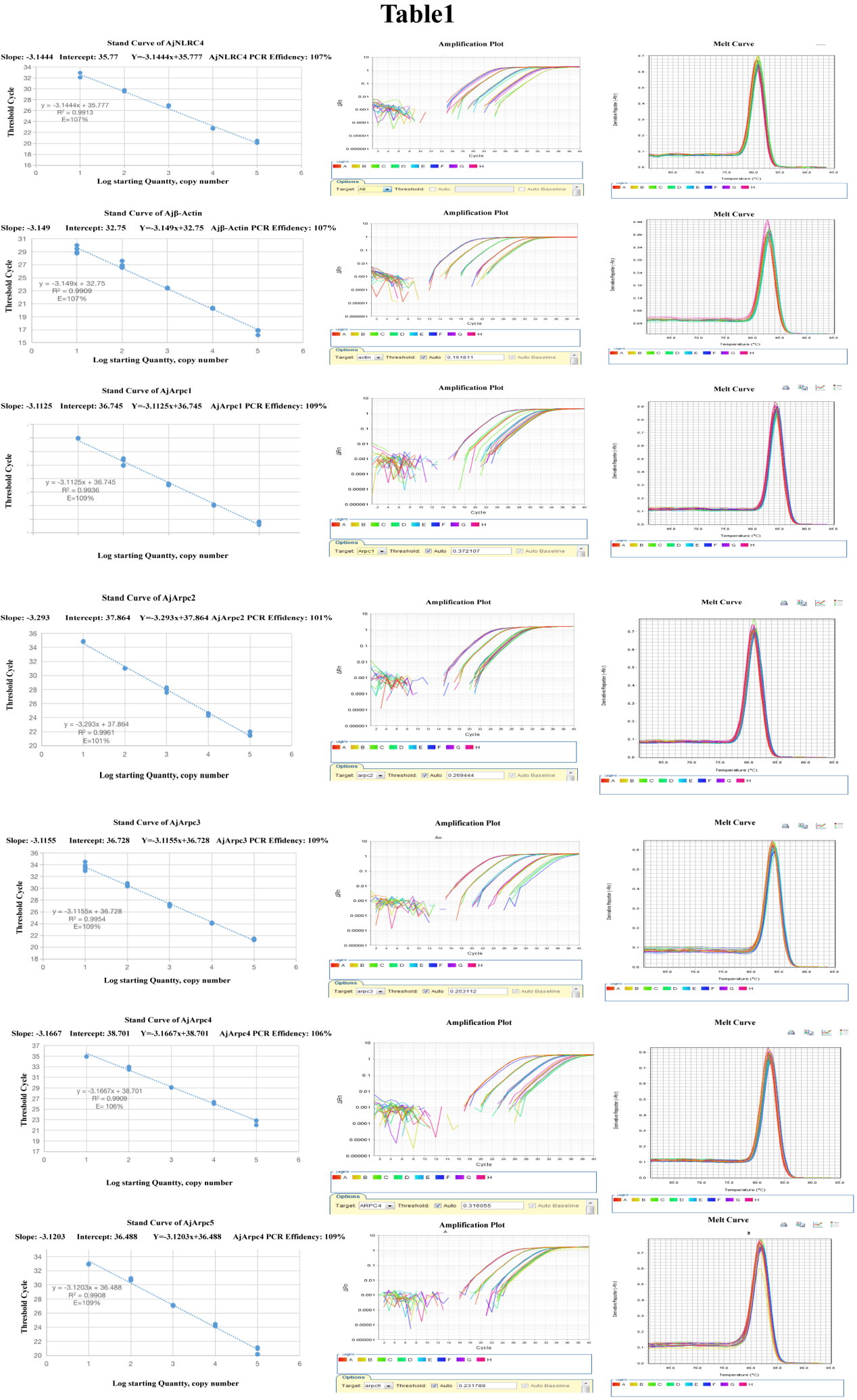


1. **DATA ANALYSIS**

The files were imported into Excel to calculate ΔC_T,_ ΔΔC_T,_ Power, STDEV and T-test


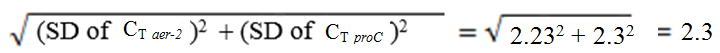
Calculated as follows：

ΔC_T_ = C_T_ _target_ – C_T_ _reference_

*_
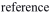

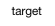
_*The standard deviation of ΔC_T_ (ΔC_T_ SD) =

ΔΔC_T_ = ΔC_T_ _sample_ – ΔC_T_ _calibrator_

Fold change (indole/DMSO, normalized by *proC*) = 2^-ΔΔCT^

1. **All qPCR data in this experiment are shown in the following table**

**Fig 4C.** The cycling numbers and fold change of *AjANLRC4* in AjNLRC4-RNAi

| **Gene** | ***β-Actin*(reference gene)** | | | ***ΑjNLRC4*** | |
| --- | --- | --- | --- | --- | --- |
| **Treatment** | NC | AjNLRC4-RNAi | | NC | AjNLRC4-RNAi |
| C_T_ 1 | 21.33 | 21.65 | | 26.21 | 27.58 |
| C_T_ 2 | 21.36 | 21.66 | | 26.17 | 27.82 |
| C_T_ 3 | 22.48 | 21.92 | | 27.04 | 27.90 |
| C_T_ Mean | 21.72 ± 0.656 | 21.74± 0.157 | | 26.47± 0.48 | 27.77±0.165 |
| ΔC_T_ |  |  | | 4.75±0.171 | 6.025±0.123 |
| ΔΔC_T_ |  | |  | 1.27435 | |
| Fold change |  | | | 0.413 | |

**Fig 6A.** The cycling numbers and fold change of *AjANLRC4* in AjNLRC4 overexpression

| Gene | ***β-Actin(reference gene)*** | | | ***ΑjNLRC4*** | |
| --- | --- | --- | --- | --- | --- |
| Treatment | Tex-His-tag | AjNLRC4-over | | Tex-His-tag | AjNLRC4-over |
| CT 1 | 20.89 | 20.53 | | 28.24 | 25.84 |
| CT 2 | 20.93 | 20.73 | | 27.48 | 25.94 |
| CT 3 | 20.98 | 20.92 | | 27.99 | 26.07 |
| CT Mean | 20.93 ± 0.04 | 20.72± 0.19 | | 27.909± 0.38 | 25.954±0.119 |
| ΔCT |  |  | | 6.97±0.40 | 5.22±0.014 |
| ΔΔCT |  | |  | -1.746 | |
| Fold change |  | | | 3.3536 | |

**Fig 8E.** The cycling numbers and fold change of *AjArpc1* in AjNLRC4-RNAi

| Gene | ***β-Actin(reference gene)*** | | | ***ΑjArpc1*** | |
| --- | --- | --- | --- | --- | --- |
| Treatment | NC | AjNLRC4-RNAi | | NC | AjNLRC4-RNAi |
| CT 1 | 17.74 | 17.66 | | 21.94 | 21.86 |
| CT 2 | 16.93 | 17.10 | | 21.41 | 21.64 |
| CT3 | 16.94 | 17.51 | | 21.26 | 21.63 |
| CT Mean | 17.20 ± 0.46 | 17.43 ± 0.29 | | 21.54 ± 0.358 | 21.71 ± 0.13 |
| ΔCT |  |  | | 4.33 ± 0.14 | 4.28 ± 0.22 |
| ΔΔCT |  | |  | -0.05 | |
| Fold change |  | | | 1.036 | |

**Table 8E.** The cycling numbers and fold change of *AjArpc2* in AjNLRC4-RNAi

| Gene | ***β-Actin(reference gene)*** | | | ***ΑjArpc2*** | |
| --- | --- | --- | --- | --- | --- |
| Treatment | NC | AjNLRC4-RNAi | | NC | AjNLRC4-RNAi |
| CT 1 | 17.74 | 17.66 | | 23.66617775 | 23.3453598 |
| CT 2 | 16.93 | 17.10 | | 22.92048645 | 23.04699135 |
| CT 3 | 16.94 | 17.51 | | 22.96751022 | 23.29893494 |
| CT Mean | 17.20± 0.46 | 17.43 ± 0.29 | | 23.23± 0.16 | 23.18 ± 0.41 |
| ΔCT |  |  | | 5.97±0.04 | 5.79±0.13 |
| ΔΔCT |  | |  | -0.17 | |
| Fold change |  | | | 1.13 | |

**Table 8E.** The cycling numbers and fold change of *AjArpc3* in AjNLRC4-RNAi

| **Gene** | ***β-Actin*(reference gene)** | | | ***ΑjArpc3*** | |
| --- | --- | --- | --- | --- | --- |
| **Treatment** | NC | AjNLRC4-RNAi | | NC | AjNLRC4-RNAi |
| C_T_ 1 | 17.74 | 17.66 | | 26.77 | 26.96 |
| C_T_ 2 | 16.93 | 17.10 | | 26.59 | 26.92 |
| C_T_ 3 | 16.94 | 17.51 | | 26.31 | 26.75 |
| C_T_ Mean | 17.20 ± 0.46 | 17.43± 0.29 | | 26.56±0.23 | 26.88±0.11 |
| ΔC_T_ |  |  | | 9.35±0.31 | 9.35±0.31 |
| ΔΔC_T_ |  | |  | 0.09 | |
| Fold change |  | | | 0.93 | |

**Table8E.** The cycling numbers and fold change of *AjArpc4* in AjNLRC4-RNAi

| **Gene** | ***β-Actin*(reference gene)** | | | ***ΑjArpc4*** | |
| --- | --- | --- | --- | --- | --- |
| **Treatment** | NC | AjNLRC4-RNAi | | NC | AjNLRC4-RNAi |
| C_T_ 1 | 22.48 | 22.38 | | 25.41 | 28.20 |
| C_T_ 2 | 22.52 | 22.92 | | 26.42 | 28.74 |
| C_T_ 3 | 23.61 | 22.97 | | 26.96 | 28.93 |
| C_T_ Mean | 22.87 ± 0.64 | 22.76± 0.328 | | 26.27± 0.78 | 28.63±0.37 |
| ΔC_T_ |  |  | | 3.39±0.48 | 5.87±0.08 |
| ΔΔC_T_ |  | |  | 2.47 | |
| Fold change |  | | | 0.18 | |

**Table 8E.** The cycling numbers and fold change of *AjArpc5* in AjNLRC4-RNAi

| **Gene** | ***β-Actin*(reference gene)** | | | ***ΑjArpc5*** | |
| --- | --- | --- | --- | --- | --- |
| **Treatment** | NC | AjNLRC4-RNAi | | NC | AjNLRC4-RNAi |
| C_T_ 1 | 21.33 | 21.65 | | 26.21 | 27.58 |
| C_T_ 2 | 21.36 | 21.66 | | 26.17 | 27.82 |
| C_T_ 3 | 22.48 | 21.92 | | 27.04 | 27.90 |
| C_T_ Mean |  |  | | 25.72± 3.6 | 26.53±3.02 |
| ΔC_T_ |  |  | | 4.15±0.12 | 5.14±0.16 |
| ΔΔC_T_ |  | |  | 0.98 | |
| Fold change |  | | | 0.52 | |

**Table 8F.** The cycling numbers and fold change of *AjArpc4* in CK666 treated

| **Gene** | ***β-Actin*(reference gene)** | | | ***ΑjArpc4*** | |
| --- | --- | --- | --- | --- | --- |
| **Treatment** | Untreated | CK666 treated | | Untreated | CK666 treated |
| C_T_ 1 | 18.83 | 14.90 | | 24.13 | 22.81 |
| C_T_ 2 | 18.94 | 14.76 | | 24.47 | 22.96 |
| C_T_ 3 | 18.82 | 14.69 | | 24.20 | 22.97 |
| C_T_ Mean | 18.86 ± 0.06 | 14.7897± 0.10 | | 24.27± 0.08 | 22.91 ± 0.08 |
| ΔC_T_ |  |  | | 5.40 ± 0.12 | 8.12 ± 0.19 |
| ΔΔC_T_ |  | |  | 2.72 | |
| Fold change |  | | | 0.15 | |

**Table 8G.** The cycling numbers and fold change of *AjArpc4* in *V.s* treated different times

| **Gene** | ***β-Actin*(reference gene)** | | | | | | ***ΑjArpc4*** | | | | | |
| --- | --- | --- | --- | --- | --- | --- | --- | --- | --- | --- | --- | --- |
| **Treatment** | Untreated | V.s 6h | V.s 12h | V.s 24h | V.s 24h | V.s 72h | Untreated | V.s 6h | V.s 12h | V.s 24h | V.s 48h | V.s 72h |
| C_T_ 1 | 19.32 | 17.87 | 18.00 | 18.80 | 17.52 | 18.42 | 30.62 | 25.41 | 26.99 | 28.41 | 28.50 | 26.86 |
| C_T_ 2 | 19.00 | 20.30 | 18.56 | 18.40 | 18.41 | 18.20 | 29.72 | 29.02 | 25.31 | 27.83 | 27.67 | 25.92 |
| C_T_ 3 | 18.68 | 17.78 | 17.75 | 18.22 | 16.92 | 18.47 | 30.08 | 25.89 | 25.98 | 26.88 | 26.73 | 25.29 |
| Fold change |  | | | | | | 1 | 8.08 | 8.94 | 3.75 | 2.24 | 11.18 |

Table 8G. The cycling numbers and fold change of *AjArpc5* in *V.s* treated different times

| **Gene** | ***β-Actin*(reference gene)** | | | | | | ***ΑjArpc5*** | | | | | |
| --- | --- | --- | --- | --- | --- | --- | --- | --- | --- | --- | --- | --- |
| **Treatment** | Untreated | V.s 6h | V.s 12h | V.s 24h | V.s 24h | V.s 72h | Untreated | V.s 6h | V.s 12h | V.s 24h | V.s 48h | V.s 72h |
| C_T_ 1 | 19.32 | 17.87 | 18.00 | 18.80 | 17.52 | 18.42 | 25.73 | 23.75 | 25.09 | 25.07 | 24.43 | 24.45 |
| C_T_ 2 | 19.00 | 20.30 | 18.56 | 18.40 | 18.41 | 18.20 | 25.14 | 24.89 | 24.71 | 25.56 | 24.93 | 24.89 |
| C_T_ 3 | 18.68 | 17.78 | 17.75 | 18.22 | 16.92 | 18.47 | 24.89 | 24.28 | 24.39 | 24.74 | 23.51 | 24.48 |
| Fold change |  | | | | | | 1 | 1.51 | 0.77 | 0.76 | 0.74 | 1.00 |

**Fig 8.** The cycling numbers and fold change of *AjArpc4* in AjArpc4-RNAi

| **Gene** | ***β-Actin*(reference gene)** | | | ***ΑjArpc4*** | |
| --- | --- | --- | --- | --- | --- |
| **Treatment** | Untreated | CK666 treated | | Untreated | CK666 treated |
| C_T_ 1 | 22.92 | 21.95 | | 28.83 | 29.60 |
| C_T_ 2 | 22.96 | 21.88 | | 28.93 | 28.55 |
| C_T_ 3 | 23.78 | 21.74 | | 28.63 | 28.94 |
| C_T_ Mean | 23.23 ± 2.23 | 21.86± 1.7 | | 28.8± 0.15 | 29.03±0.53 |
| ΔC_T_ |  |  | | 5.57±0.50 | 7.17±0.11 |
| ΔΔC_T_ |  | |  | 1.59 | |
| Fold change |  | | | 0.33 | |

**Supporting figures**

^
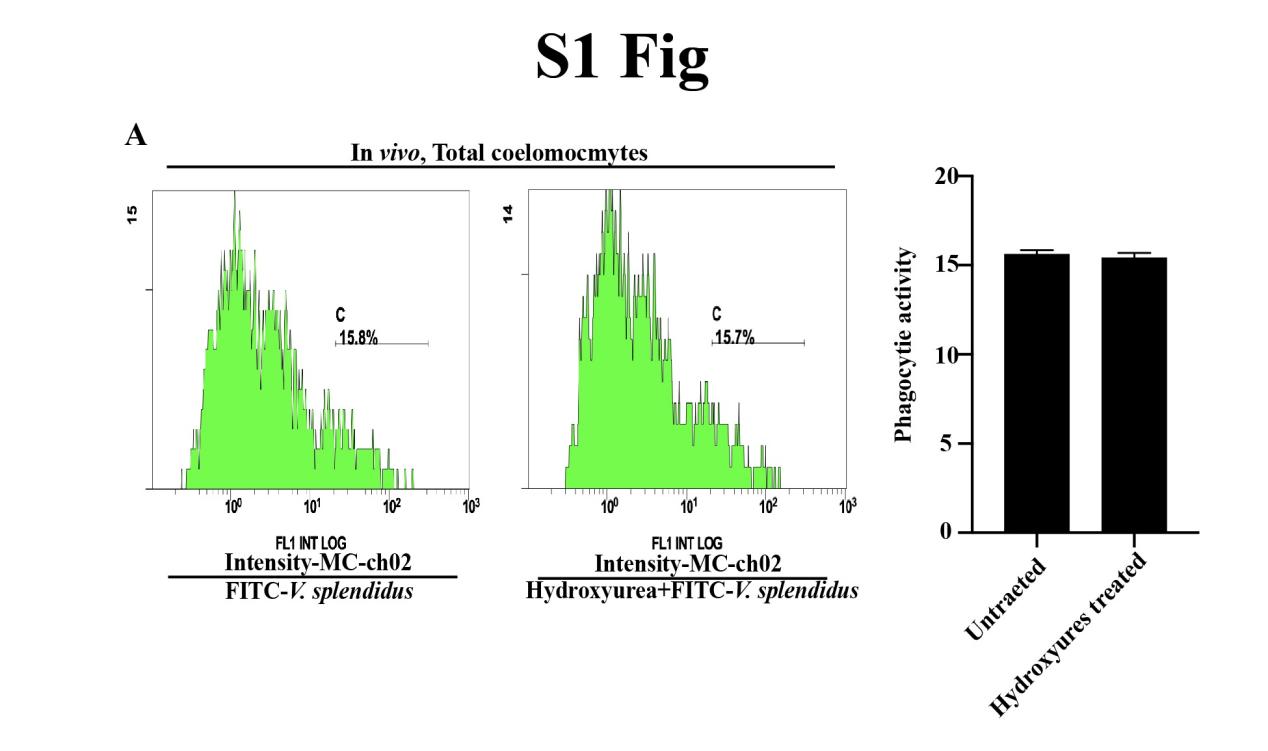
^

^
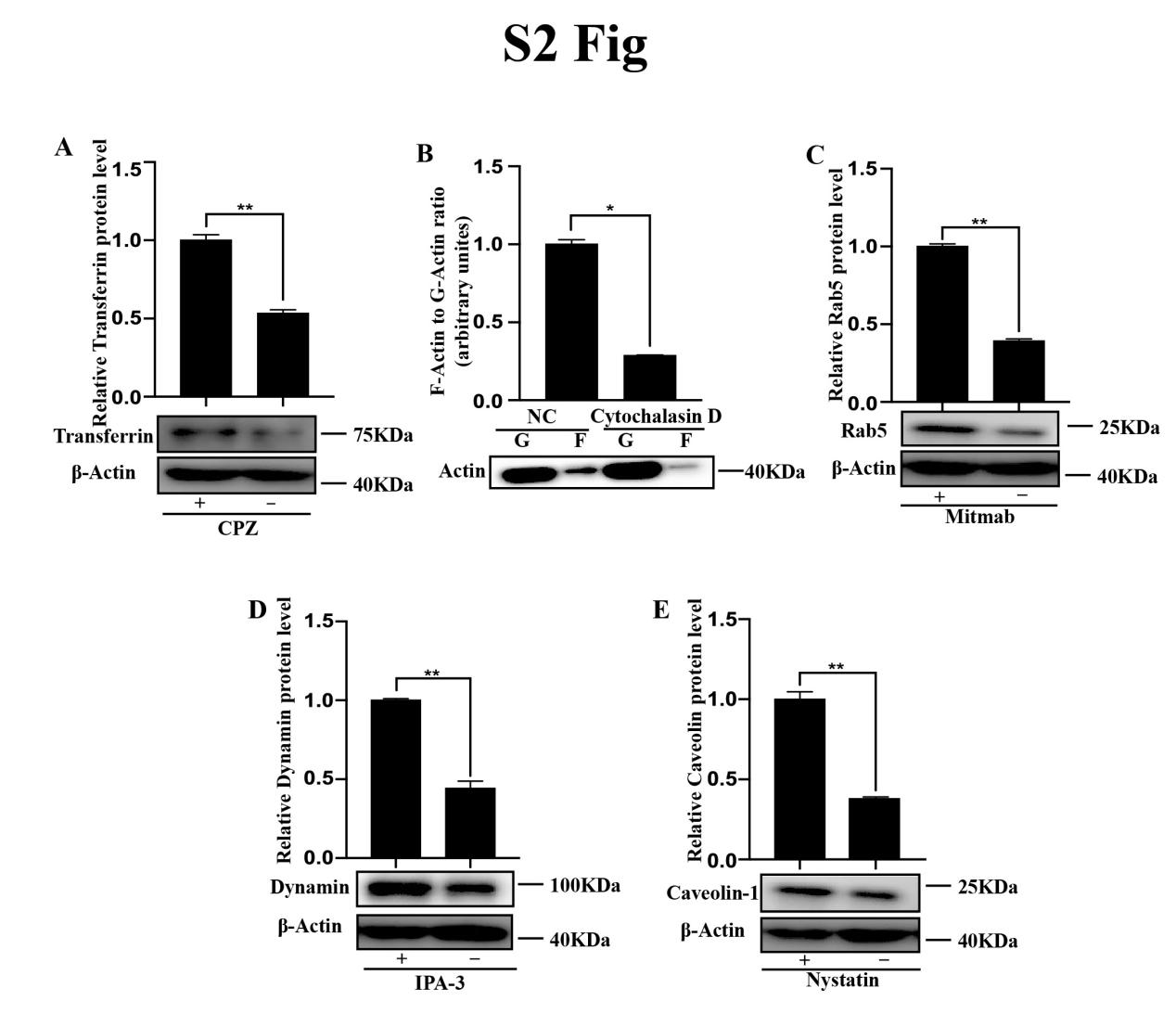
^

^
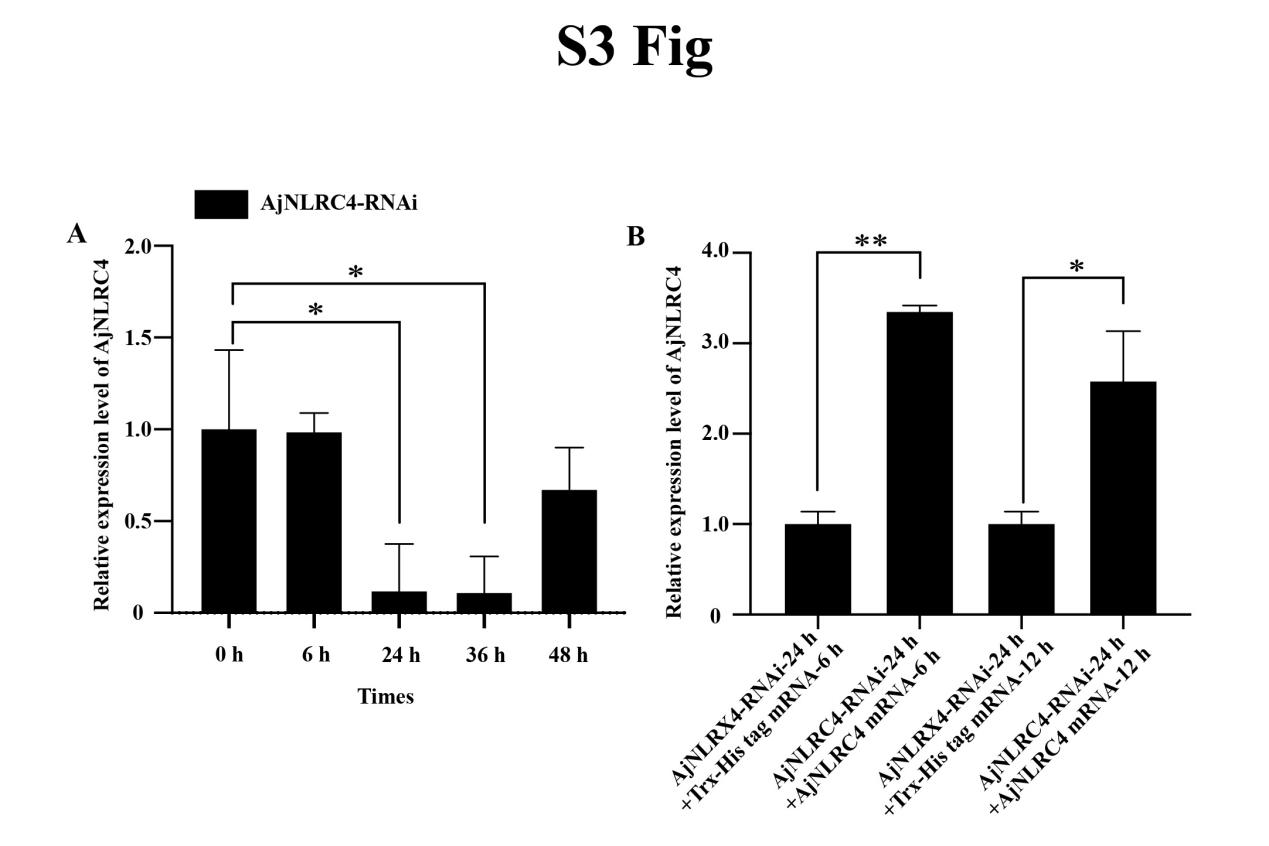
^
