# Supplementary figures and images for: A unique NLRC4 receptor from echinoderms mediates Vibrio phagocytosis via rearrangement of the cytoskeleton and polymerization of F-actin
Source: PLoS Pathog. 2021 Dec 13;17(12):e1010145. doi: 10.1371/journal.ppat.1010145 (PMC8699970; doi:10.1371/journal.ppat.1010145)

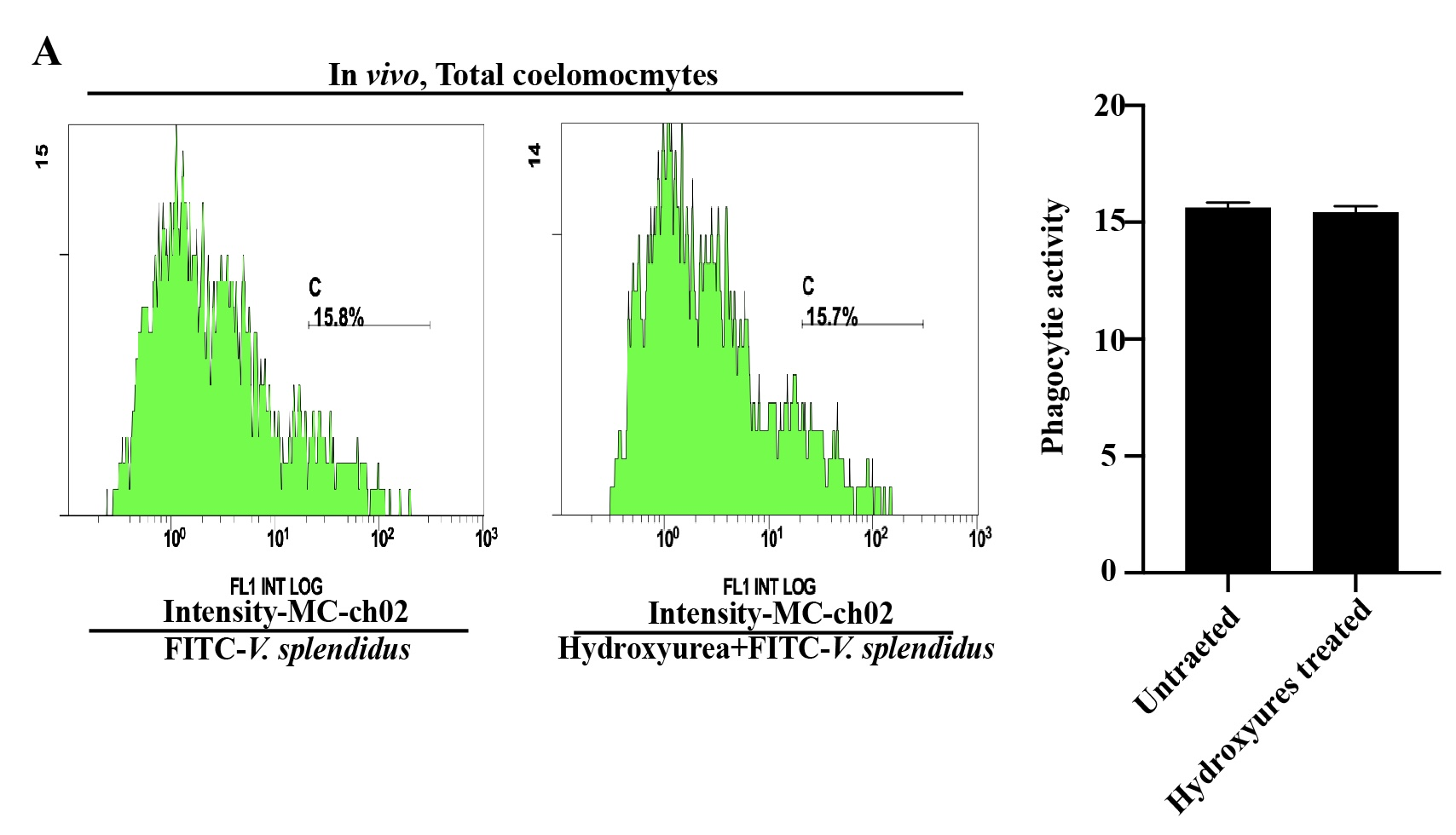

Supplement: S1 Fig — 200 μg Hydroxyurea solution was injected into sea cucumbers for 6 h before FITC-labeled V. splendidus injection. Same volume of PBS were served as a control. The phagocytic activity was determined by flow cytometry. The graphs are representative of three independent assays, and the proportions were calculated from those three assays. (TIF) [file ppat.1010145.s002.tif]

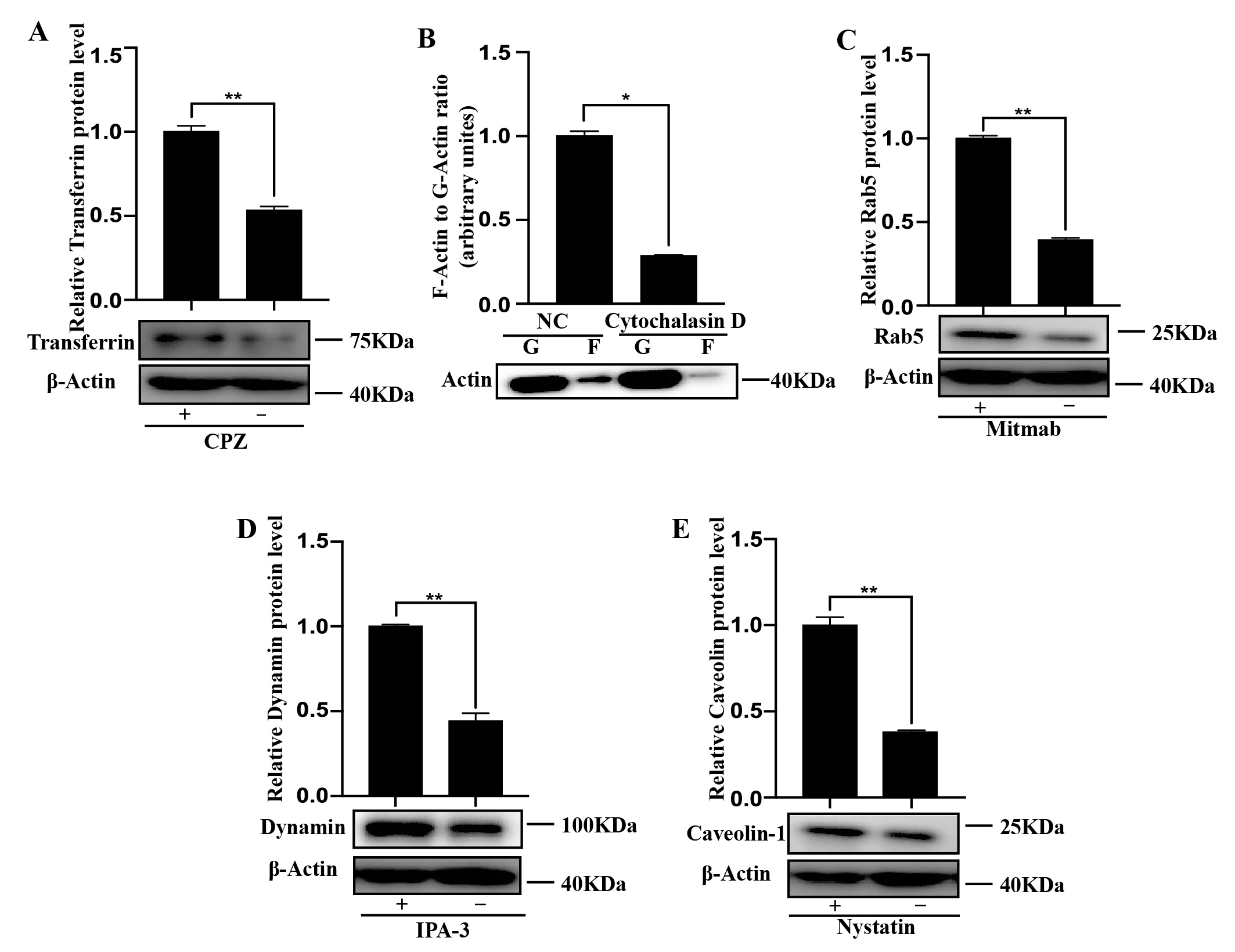

Supplement: S2 Fig — (A) The relative protein level of transferrin after CPZ inhibitor treatment. The graphs are representative of three independent assays, and the proportions were calculated from those three assays, *p < 0.05. (B) The ratio of G-Actin/F-Actin after cytochalasin D inhibitor treatmen. The graphs are representative of three independent assays, and the proportions were calculated from those three assays, *p < 0.05. (C) The relative protein level of Rab5 after Mitmab inhibitor treatment. The graphs are representative of three independent assays, and the proportions were calculated from those three assays, *p < 0.05. (D) The relative protein level of dynamin after IPA-3 inhibitor treatment.The graphs are representative of three independent assays, and the proportions were calculated from those three assays, *p < 0.05. (E) The relative protein level of caveolin1 after nystatin inhibitor treatment. The graphs are representative of three independent assays, and the proportions were calculated from those three assays, *p < 0.05. (TIF) [file ppat.1010145.s003.tif]

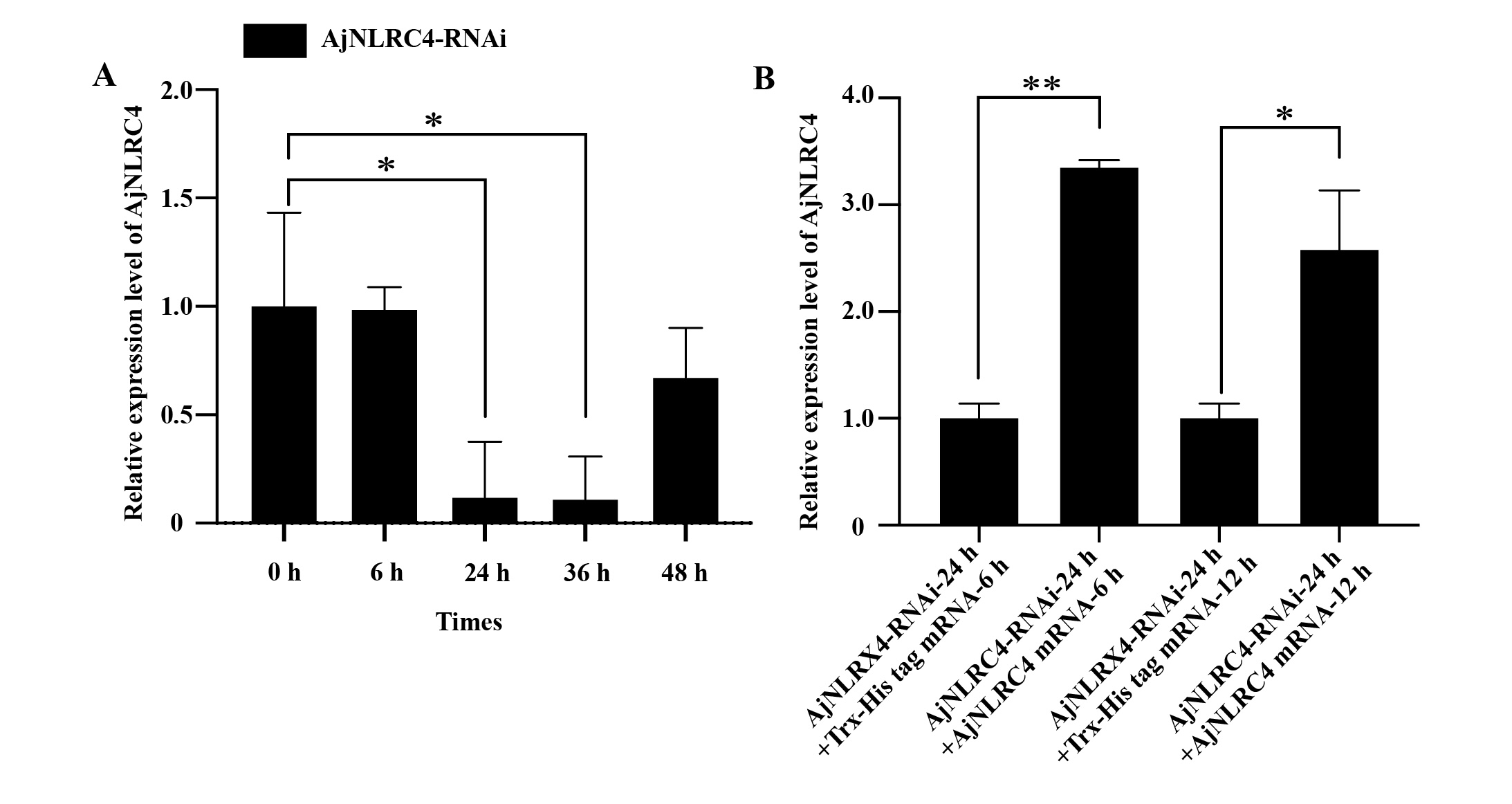

Supplement: S3 Fig — (A) The efficiency of AjNLRC4-RNAi in coelomocytes at different times was determined using qPCR. The graphs are representative of three independent assays, and the proportions were calculated from those three assays, *p < 0.05, **p < 0.01. (B) The relative expression of AjNLRC4 mRNA in the rescue experiment. The graphs are representative of three independent assays, and the proportions were calculated from those three assays, *p < 0.05. (TIF) [file ppat.1010145.s004.tif]
